# Achieved systolic blood pressure and cardiovascular outcomes in advanced chronic kidney disease

**DOI:** 10.1093/ckj/sfag296

**Published:** 2026-08-31

**Authors:** Ehab Al-Sodany, Karolina Szummer, Joakim Österman, Caroline E Dietrich, Juan-Jesus Carrero, Olof Heimbürger, Peter Bárány, Marie Evans

**Affiliations:** Renal Unit, Department of Clinical Intervention and Technology (CLINTEC), Karolinska Institutet, Stockholm Sweden; Department of Medicine Huddinge, Karolinska Institutet, Stockholm, Sweden; Renal Unit, Department of Clinical Intervention and Technology (CLINTEC), Karolinska Institutet, Stockholm Sweden; Division of Clinical Epidemiology, Department of Medicine Solna, Karolinska Institutet, Stockholm, Sweden; Department of Medical Epidemiology and Biostatistics, Karolinska Institutet, Stockholm, Sweden; Division of Nephrology, Department of Clinical Sciences, Karolinska Institutet, Danderyd Hospital, Stockholm, Sweden; Renal Unit, Department of Clinical Intervention and Technology (CLINTEC), Karolinska Institutet, Stockholm Sweden; Renal Unit, Department of Clinical Intervention and Technology (CLINTEC), Karolinska Institutet, Stockholm Sweden; Renal Unit, Department of Clinical Intervention and Technology (CLINTEC), Karolinska Institutet, Stockholm Sweden

**Keywords:** cardiovascular disease, chronic kidney disease, hypertension, observational cohort, systolic blood pressure

## Abstract

**Background:**

In advanced chronic kidney disease (CKD) evidence for lower systolic blood pressure (SBP) targets is limited and guideline recommendations are conflicting. We investigated the association between achieved SBP and major adverse cardiovascular events (MACE) in nephrology-referred patients with CKD.

**Methods:**

In this nationwide, observational cohort from the Swedish Renal Register–chronic kidney disease (SRR-CKD), we included adults with CKD stages 3–5 referred to nephrology outpatient clinics between 2006 and 2017. Mean office SBP during the first year was categorized in incrementally higher SBP groups with 130–<140 mmHg as reference. The primary outcome was MACE; secondary outcomes were its components and all-cause death, analyzed using cause-specific multivariable-adjusted flexible parametric survival models.

**Results:**

Among 23 317 patients (median age 72 years; 64% men; median estimated glomerular filteration rate (eGFR) 24 ml/min/1.73 m²), over a median follow-up of 2.5 years, 8556 (37%) experienced MACE and 7686 (33%) died. Compared with SBP 130–<140 mmHg, SBP 120–<130 mmHg was associated with lower risks of MACE [adjusted hazard ratio (HR) 0.93, 95% confidence interval (CI) 0.87–0.99] and all-cause mortality (HR 0.93, 95% CI 0.87–0.99), whereas SBP ≥160 mmHg was associated with higher risks of MACE (HR 1.38, 95% CI 1.28–1.49) and death (HR 1.29, 95% CI 1.20–1.38). MACE risk was nonproportional and declined over time in patients with SBP 100–<120 mmHg, demonstrating lower MACE risk compared to 130–<140 after 3 years. In continuous analyses, MACE risk was lowest around 125 mmHg and increased at higher SBP levels.

**Conclusions:**

In nephrology-referred patients with advanced CKD, achieved SBP around 120–<130 mmHg (near 125 mmHg) was associated with the lowest cardiovascular and mortality risk, while SBP ≥140 mmHg, especially ≥160 mmHg, was consistently associated with higher MACE risk.

## Graphical Abstract

**Graphical Abstract:**
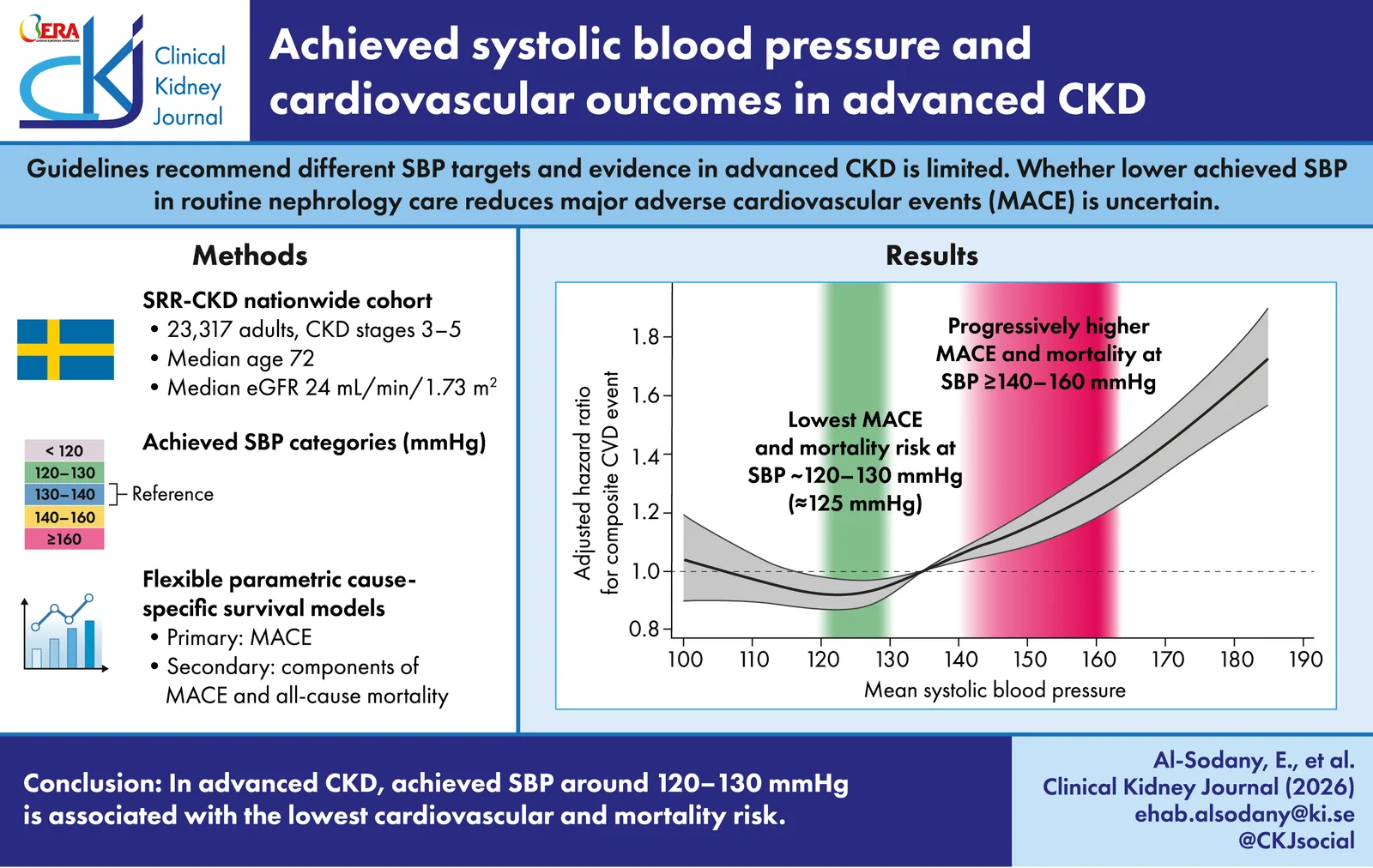

KEY LEARNING POINTS **What was known:**

- Hypertension is a key modifiable risk factor for CKD progression and adverse cardiovascular outcomes.
- Intensive SBP lowering reduces cardiovascular events in selected high-risk populations, but the evidence base for intensive targets in advanced CKD is limited and heterogeneous.
- Patients with marked albuminuria, diabetes, polycystic kidney disease, glomerulonephritis or very low eGFR often were not included in major BP trials, limiting generalizability to real-world nephrology populations.

**This study adds:**

- In a nationwide cohort of 23 317 nephrology-referred patients with predominantly stage 3–5 CKD, the lowest predicted risk of MACE was observed at an achieved SBP around 125 mmHg, with increased risks at higher SBP levels.
- SBP 120–<130 mmHg was associated with lower risks of both MACE and all-cause mortality compared with the standard SBP range of 130–<140 mmHg.
- SBP ≥140 mmHg, and particularly ≥160 mmHg, was consistently associated with higher short- and longer-term risks of MACE, the composite of MACE or death, and all-cause mortality.
- Stable patients with SBP 100–<120 mmHg who survived at least one year had lower 3-year risks of MACE and of MACE or death than those with SBP 130–<140 mmHg, but no clear additional average benefit below 120 mmHg relative to 125 mmHg.
- These associations were broadly consistent across age, sex, diabetes status, comorbidity burden, and albuminuria categories, and were robust in multiple sensitivity analyses, including restriction to eGFR <30 ml/min/1.73 m².

**Potential impact:**

- SBP management toward lower achieved levels, particularly around 120–<130 mmHg, may help reduce cardiovascular morbidity and mortality in patients with advanced CKD managed in routine nephrology care.
- The findings support guideline strategies that aim for SBP below 130–140 mmHg when tolerated, but also highlight the importance of individualized BP targets and careful assessment of very low SBP, especially in older, frail, or unstable patients who may not have been represented in major trials.

## INTRODUCTION

Hypertension is a key modifiable risk factor for both chronic kidney disease (CKD) and cardiovascular disease (CVD) [1]. In recent decades, randomized controlled trials and meta-analyses have demonstrated that lower blood pressure (BP) targets reduce the risk of cardiovascular events and mortality in high-risk populations [2, 3]. The Systolic blood Pressure Intervention Trial (SPRINT) is especially influential, showing significant reductions in the composite endpoint of CVD events and death with a systolic blood pressure (SBP) target of <120 mmHg compared with a standard target <140 mmHg [4]. The SPRINT findings strongly influenced the 2021 Kidney Disease Improving Global Outcomes (KDIGO) guideline, which recommends lowering the SBP to <120 mmHg when tolerated, using standardized office BP measurements in adults with CKD [5]. This KDIGO recommendation marked a dramatic shift in practice, but it was predominantly based on a post–hoc subgroup analysis of SPRINT participants with CKD, in which neither the primary cardiovascular outcome nor the pre–specified composite kidney outcome were significantly reduced with intensive BP lowering [6]. Consequently, many experts have contended that the quality of evidence supporting such a profound paradigm shift was insufficient, raising questions about the validity and generalizability of a universal <120 mmHg target in CKD [7].

By contrast, the 2023 European Society of Hypertension (ESH) guideline—endorsed by the European Renal Association (ERA)—recommends lowering office BP to <140/90 mmHg in all patients with CKD, with intensification to <130/80 mmHg in most patients if tolerated, while explicitly advising against targets <120/70 mmHg [8]. These divergent recommendations illustrate the limited and partly conflicting evidence base and underscore the need for additional data on BP targets in advanced CKD managed in routine clinical practice.

However, significant knowledge gaps remain regarding the long-term benefits and risks of intensive BP lowering in advanced stage CKD [9, 10]. SPRINT excluded key subgroups, including patients with eGFR <20 ml/min/1.73 m², diabetes, glomerulonephritis, polycystic kidney disease, and marked albuminuria resulting in a study population with relatively preserved kidney function and only about one–third having CKD [4]. Furthermore, older BP trials in CKD lacked strict BP targets [11–13] and existing observational studies are limited by scope [14–17], size [11, 18–21], or disease specificity [22, 23]. Some observational data also suggest a J-shaped association between SBP and CVD outcomes, raising concern about potential harms of excessive low BP lowering in CKD [24, 25]. Considering these uncertainties, KDIGO emphasizes the need for high quality RCTs specifically addressing intensive BP control in CKD patients with diabetes, significant proteinuria, or severely reduced eGFR. In the absence of such trials, there is a need for robust real–world data that mirror the complexity of patients seen in nephrology clinics. We therefore aimed to expand on SPRINT findings by mimicking key aspects of its design in an observational setting. Specifically, we investigated whether lower achieved SBP is associated with reduced CVD risk in a large, unselected cohort of nephrology-referred patients with stage 3–5 CKD, accounting for diverse etiologies and risk factors, including albuminuria.

## MATERIALS AND METHODS

### Study population

We used data from the Swedish Renal Register—chronic kidney disease (SRR-CKD) which includes 98% of nephrology clinics in Sweden and captures 75%–90% of referred patients with CKD stages 4–5 [26]. Patients enrolled between 2006 and 2017 without any prior KRT were eligible (*n* = 29 617).

The index date for follow-up was set exactly 1 year after inclusion, and information on SBP and other variables was collected during the first year (Supplementary Fig. S1). We excluded patients who initiated kidney replacement therapy (KRT), including kidney transplantation (*n* = 1106) and those who died within the first year (*n* = 2229) or lacked inclusion date at index date (*n* = 18). Additionally, we excluded 9 patients below 18 years of age, 1997 patients with eGFR ≥60 ml/min/1.73 m^2^, 607 patients with missing SBP, and 244 individuals with a mean SBP <100 mmHg as such low SBP values likely reflected comorbid conditions such as heart failure rather than treatment effects [25]. The final analytic cohort included 23 317 patients.

Patients were followed until the occurrence of an outcome event, death, emigration, or the end of the study (31 December 2017). Outcome events occurring after initiation of KRT were included in the analyses.

All procedures complied with the Declaration of Helsinki. Patients were informed of their inclusion in SRR-CKD and could opt out; formal written informed consent was not required under Swedish regulations for quality registers. The study protocol was approved by the Regional Ethics Review Committee in Stockholm (DNR 2011/618-31/4; 2014/1236-32; 2017/1891-32).

### Blood pressure measurements

The exposure was mean office SBP over the 12 months prior to the index date, calculated from all available clinic BP measurements during that period. As an example, BP measurements at Karolinska University Hospital, the largest center in Sweden, were obtained during routine outpatient nephrology visits using automated oscillometric devices (CARESCAPE V100 Monitor and Welch Allyn Connex Spot Monitor) under attended, nurse-performed conditions, with standardized rest, positioning, and cuff sizing, and typically a single reading per visit was recorded for registry purposes. For other participating nephrology clinics in the SRR CKD registry, specific device models, exact implementation of rest periods, and the use of repeated readings are not systematically recorded, and we cannot verify that the same devices or single reading approach were used. However, all clinics operate within the Swedish public healthcare system and are expected to use calibrated devices and to follow national and international guidelines for BP measurement, including those from KDIGO and the European Society of Hypertension [5, 8].

Thus, while measurement procedures may vary somewhat between centers, the BP data overall represent high quality attended clinic BP measurements that reflect real world nephrology practice. Detailed preparations at nephrology clinics and patient instructions for BP measurement are described in the Supplementary material.

### Outcomes

The primary outcome was a composite of major adverse cardiovascular events (MACE), specified as first occurrence of a nonfatal myocardial infarction, nonmyocardial infarction acute coronary syndrome, stroke, heart failure hospitalization, or death attributable to CVD (Table S1). Secondary endpoints included each individual components of the MACE endpoint and death from any cause. Outcomes were ascertained using ICD-10 from hospital admissions through linkage with the national Swedish Patient Register [27]. Information regarding KRT, including kidney transplantation, and cause of death was obtained from the SRR-CKD.

### Covariates

Demographics, body mass index, and primary kidney disease, were obtained from the SRR-CKD. Laboratory values were carried forward from the closest visit before the index date. The following laboratory values were mandated at outpatient visits and were registered in SRR–CKD when available: serum creatinine, hemoglobin, albumin, phosphate, calcium, parathyroid hormone, albuminuria, and C–reactive protein. However, for some visits not all values were measured or recorded. Albumin-to-creatinine ratio (ACR) was categorized into no albuminuria (KDIGO category A1) if ACR <3 mg/mmol, A2 if ACR was 3 to <30 mg/mmol, and A3 if ACR was ≥30 mg/mmol [5].

Information on comorbidities at the index date, was gathered from ICD-10 codes in the Swedish Patient Register (Table S2) using all available diagnoses, and medication data classified according to Anatomic Therapeutic Chemical (ATC) system, were collected from the Prescribed Drug Register (Table S3), which includes all prescribed and dispensed medications collected from any pharmacy in Sweden [28].

### Statistical analysis

Descriptive statistics including means, medians with inter-quartile ranges (IQR) and frequencies with proportions, were used to summarize baseline characteristics overall and across SBP categories. The association between SBP and MACE was examined using both categorical (100 < 120, 120 < 130, 130 < 140, 140 < 150, 150 < 160, and ≥160 mmHg) and continuous [restricted cubic spline with 4 degrees of freedom (df)] approaches in a flexible parametric proportional hazards framework to allow for nonlinear effects.

Cause-specific hazard ratios (HRs) with 95% confidence intervals (CIs) were estimated from both unadjusted and adjusted models, the latter controlled for pre-defined confounders, using 5 degrees of freedom (df) for the baseline hazard rate (see Supplement). To account for time-varying effects interactions between SBP and time (3 df) were included, and HRs at 1 and 3 years were derived. For comparability with SPRINT, an SBP of 125 mmHg was used as the reference in continuous analyses of SBP. We also compared our CKD cohort with SPRINT CKD participants to contextualize findings (Table S4). To complement relative rate estimates we calculated the cause-specific cumulative incidence function (CIF) for MACE, accounting for the competing risk of noncardiovascular deaths. This was done nonparametrically using the Aalen-Johansen estimator and predicted through a model-based approach using a flexible parametric cause-specific survival model adjusted as in the primary analysis (see Supplement). Predictions from the model-based CIF used mean or reference values for covariates. We also assessed potential interaction between SBP and pre-defined subgroups including age (< or ≥65 years), sex, diabetes, albuminuria category, ACEi/ARB use, and Charlson Comorbidity index (<4 or ≥4) using likelihood ratio tests in the full primary outcome model.

Several sensitivity analyses were conducted. First, we restricted analyses to those with eGFR <30 ml/min/1.73 m^2^ at index date (*n* = 16 822). Second, we adjusted for albuminuria where available, and we additionally computed an analysis accounting for missing albuminuria measurements as a categorical variable. Third, we performed two landmark analyses including patients with minimum follow-up until 6 months, and 12 months with at least two follow-up visits. Lastly, we analyzed all patients >18 years of age with eGFR <60 ml/min/1.73 m², including also patients with SBP <100 mmHg and patients who started KRT or died within 12 months using their *first* SBP recording (*n* = 28 113), and additionally used kidney replacement therapy as a time-dependent covariate. All statistical tests were two-sided with a 5% significance level and conducted using stmerlin and multistate in Stata 18 (StataCorp LLC) [29–31].

## RESULTS

### Baseline characteristics

At index date, the median age of our main cohort was 72 years (IQR 63–80), with 64% being men. The median eGFR was 24 ml/min/1.73 m² (IQR 17.4, 30.6). Among patients with available albuminuria data, median ACR was 30.6 mg/mmol (IQR 5.6–137.0). Median SBP was 138 mmHg (IQR 127–150), and diastolic BP (DBP) was 78 mmHg (IQR 70–83) (Table 1). Applying the main inclusion and exclusion criteria for SPRINT, only 3323 patients (14%) would have been potentially eligible.

**Table 1:** Baseline characteristics of all participants and by systolic blood pressure category.

Patient characteristics missing [%]	All (*n* = 23 317)	SBP 100–120 (*n* = 2707)	SBP 120–130 (*n* = 4149)	SBP 130–140 (*n* = 5378)	SBP 140–150 (*n* = 4929)	SBP 150–160 (*n* = 2943)	SBP > 160 (*n* = 3211)
Age, years, median (IQR) [0%]	72.8 (63.7; 80.2)	72.4 (62.0; 80.2)	71.8 (61.5; 79.5)	71.9 (62.5; 79.5)	73.4 (64,6; 80.4)	73.5 (65.2; 80.5)	74.4 (65.8; 81.2)
Women, *n* (%) [0%]	8513 (36.5%)	1054 (38.9%)	1532 (36.9%)	1864 (34.7%)	1766 (35.8%)	1066 (36.2%)	1231 (38.3%)
Primary renal disease, *n* (%) [0%]							
ADPKD	1091 (4.7%)	110 (4.1%)	204 (4.9%)	303 (5.6%)	246 (5.0%)	118 (4.0%)	110 (3.4%)
Diabetic nephropathy	4824 (20.7%)	397 (14.7%)	720 (17.4%)	1040 (19.3%)	1075 (21.8%)	726 (24.7%)	866 (27.0%)
Glomerulonephritis	2141 (9.2%)	297 (11.0%)	440 (10.6%)	564 (10.5%)	429 (8.7%)	226 (7.7%)	185 (5.8%)
Hypertension	5614 (24.1%)	543 (20.1%)	906 (21.8%)	1250 (23.2%)	1224 (24.8%)	792 (26.9%)	899 (28.0%)
Pyelonephritis	689 (3.0%)	70 (2.6%)	137 (3.3%)	154 (2.9%)	147 (3.0%)	81 (2.8%)	100 (3.1%)
Renovascular disease	174 (0.8%)	18 (0.7%)	26 (0.6%)	34 (0.6%)	43 (0.9%)	23 (0.8%)	30 (0.9%)
Uremia unspecified	3956 (17.0%)	542 (20.0%)	795 (19.2%)	917 (17.1%)	790 (16.0%)	413 (14.0%)	499 (15.5%)
Other	4818 (20.7%)	730 (27.0%)	919 (22.2%)	1114 (20.7%)	970 (19.7%)	564 (19.2%)	521 (16.2%)
Missing/Unknown	10 (0.0%)	0 (0.0%)	2 (0.1%)	2 (0.0%)	5 (0.1%)	0 (0.0%)	1 (0.0%)
Charlson index, median (IQR) [0%]	4.0 (3.0; 6.0)	4.0 (3.0; 6.0)	4.0 (3.0; 6.0)	4.0 (2.0; 6.0)	4.0 (3.0; 6.0)	5.0 (3.0; 6.0)	5.0 (3.0; 6.0)
Comorbidities, *n* (%) [0%]							
Myocardial infarction, *n* (%)	5744 (24.7%)	799 (29.6%)	1083 (26.2%)	1249 (23.3%)	1171 (23.8%)	682 (23.3%)	760 (23.7%)
Diabetes mellitus, *n* (%)	9509 (40.9%)	954 (35.3%)	1530 (37.0%)	2125 (39.6%)	2089 (42.5%)	1324 (45.1%)	1487 (46.5%)
Congestive heart failure, *n* (%)	6093 (26.2%)	1003 (37.1%)	1168 (28.2%)	1267 (23.6%)	1149 (23.4%)	701 (23.9%)	805 (25.2%)
Peripheral artery disease, *n* (%)	2454 (10.6%)	316 (11.7%)	408 (9.9%)	525 (9.8%)	486 (9.9%)	332 (11.3%)	387 (12.1%)
Cerebrovascular disease, *n* (%)	4356 (18.7%)	483 (17.9%)	766 (18.5%)	970 (18.1%)	905 (18.4%)	582 (19.8%)	650 (20.3%)
Any cardiovascular disease, *n* (%)	9569 (41.1%)	1321 (48.9%)	1762 (42.6%)	2045 (38.1%)	1944 (39.5%)	1165 (39.7%)	1332 (41.6%)
Clinical data							
BMI, kg/m², median (IQR) [29%]	27.4 (24.2; 31.2)	26.9 (23.8; 30.8)	27.4 (24.1; 31.1)	27.4 (24.3; 31.4)	27.7 (24.6; 31.6)	27.3 (24.3; 31.2)	27.4 (24.2; 31.1)
eGFR, ml/min/1.73 m², median (IQR) [0%]	24.5 (18.4; 31.0)	26.5 (20.5; 33.6)	25.8 (19.5; 32.7)	24.8 (18.7; 31.5)	23.9 (17.8; 30.0)	23.1 (17.5; 29.2)	22.5 (16.7; 28.9)
SBP, mmHg, median (IQR) [0%]	138.0 (126.7; 150.0)	113.0 (109.0; 116.7)	125.0 (121.3; 126.7)	134.0 (130.7; 136.0)	143.0 (140.0; 145.5)	153.3 (150.0; 155.5)	167.5 (162.0; 175.3)
DBP, mmHg, median (IQR) [0%]	77.5 (70.0; 83.0)	70.0 (63.3; 75.0)	75.0 (68.8; 80.0)	77.5 (70.0; 82.0)	80.0 (72.0; 85.0)	80.0 (73.5; 86.7)	83.3 (77.0; 90.0)
Albumin, g/l, median (IQR) [3%]	37.0 (34.0; 40.0)	37.0 (34.0; 40.0)	37.0 (35.0; 40.0)	37.0 (35.0; 40.0)	37.0 (34.0; 40.0)	37.0 (34.0; 40.0)	37.0 (33.0; 39.0)
Hemoglobin, g/l, median (IQR) [2%]	123.0 (113.0; 134.0)	124.0 (114.0; 136.0)	125.0 (115.0; 135.0)	123.0 (114.0; 134.0)	123.0 (113.0; 133.0)	122.0 (112.0; 132.0)	121.0 (110.0; 132.0)
Phosphate, mmol/l, median (IQR) [10%]	1.2 (1.1; 1.4)	1.2 (1.0; 1.4)	1.2 (1.1; 1.4)	1.2 (1.1; 1.4)	1.2 (1.1; 1.4)	1.3 (1.1; 1.5)	1.3 (1.1; 1.5)
ACR, mg/g, median (IQR) [46%]	27.9 (5.5; 117.2)	7.8 (2.1; 37.1)	13.0 (3.1; 58.8)	23.0 (5.4; 91.4)	35.8 (7.2; 137.0)	53.1 (11.0; 169.0)	91.0 (18.5; 246.2)
Medications, *n* (%) [1%]							
ACEi/ARB, *n* (%)	14 983 (64.3%)	1660 (61.3%)	2606 (62.8%)	3434 (63.9%)	3209 (65.1%)	1931 (65.6%)	2143 (66.7%)
Beta-blockers, *n* (%)	15 185 (65.1%)	1611 (59.5%)	2509 (60.5%)	3380 (62.9%)	3210 (65.1%)	2087 (70.9%)	2388 (74.4%)
Calcium channel blockers, *n* (%)	13 266 (56.9%)	739 (27.3%)	1808 (43.6%)	3038 (56.5%)	3156 (64.0%)	2139 (72.7%)	2386 (74.3%)
Other antihypertensives, *n* (%)	3269 (14.0%)	91 (3.4%)	300 (7.2%)	584 (10.9%)	745 (15.1%)	621 (21.1%)	928 (28.9%)
Diuretics, *n* (%)	15 819 (67.8%)	1793 (66.2%)	2650 (63.9%)	3486 (64.8%)	3374 (68.5%)	2129 (72.3%)	2387 (74.3%)
Warfarin, *n* (%)	2973 (12.8%)	499 (18.4%)	606 (14.6%)	692 (12.9%)	552 (11.2%)	344 (11.7%)	280 (8.7%)
Statin, *n* (%)	13 043 (55.9%)	1472 (54.4%)	2335 (56.3%)	3062 (56.9%)	2821 (57.2%)	1679 (57.1%)	1674 (52.1%)

Values are *n* (%) for categorical variables and median (interquartile range) for continuous variables. Patients were categorized by 1-year mean systolic blood pressure; 130–<140 mmHg is the reference category. Missingness is shown in brackets where applicable. All numbers rounded to one decimal.

ACEi, angiotensin-converting enzyme inhibitor; ACR, urinary albumin-to-creatinine ratio; ADPKD, autosomal dominant polycystic kidney disease; ARB, angiotensin II receptor blocker; BMI, body mass index; CKD, chronic kidney disease; DBP, diastolic blood pressure; eGFR, estimated glomerular filtration rate; SBP, systolic blood pressure.

Converting UACR mg/mmol to mg/g divided by 0.113. Calcium mmol/L to mg/dl divided by 0.2495. Phosphate mmol/l to mg/dl divided by 0.3229. Hemoglobin g/l to g/dl divided by 10. Albumin g/l to g/dl divided by 10.

### Major adverse cardiovascular event MACE outcome

Over a median follow-up of 2.54 years (IQR 0.99–4.7), 8556 patients (37%) experienced MACE, including 3407 (15%) coronary events, 1851 (8%) myocardial infarctions, and 1730 cerebrovascular events (7.4%). In total, 7686 patients (33%) died. The relationship between SBP and MACE followed a J-shaped curve, with the lowest risk observed at around 125 mmHg and increasing risk beyond 130 mmHg (Fig. 1A). Compared with patients with SBP 130–<140 mmHg, SBP >150 mmHg was associated with a higher rate of MACE (Table 2), while SBP between 120 < 130 had lower rate of MACE HR 0.93; 95% CI (0.71–0.99). We observed nonproportional hazards of MACE over time for the lowest SBP category (100 < 120 mmHg). Initially, the HR was nonsignificantly above 1.0, but by year three, it was 19% lower than the reference group (HR 0.81; 95% CI 0.72–0.92) (Table 2).

**Figure 1:**
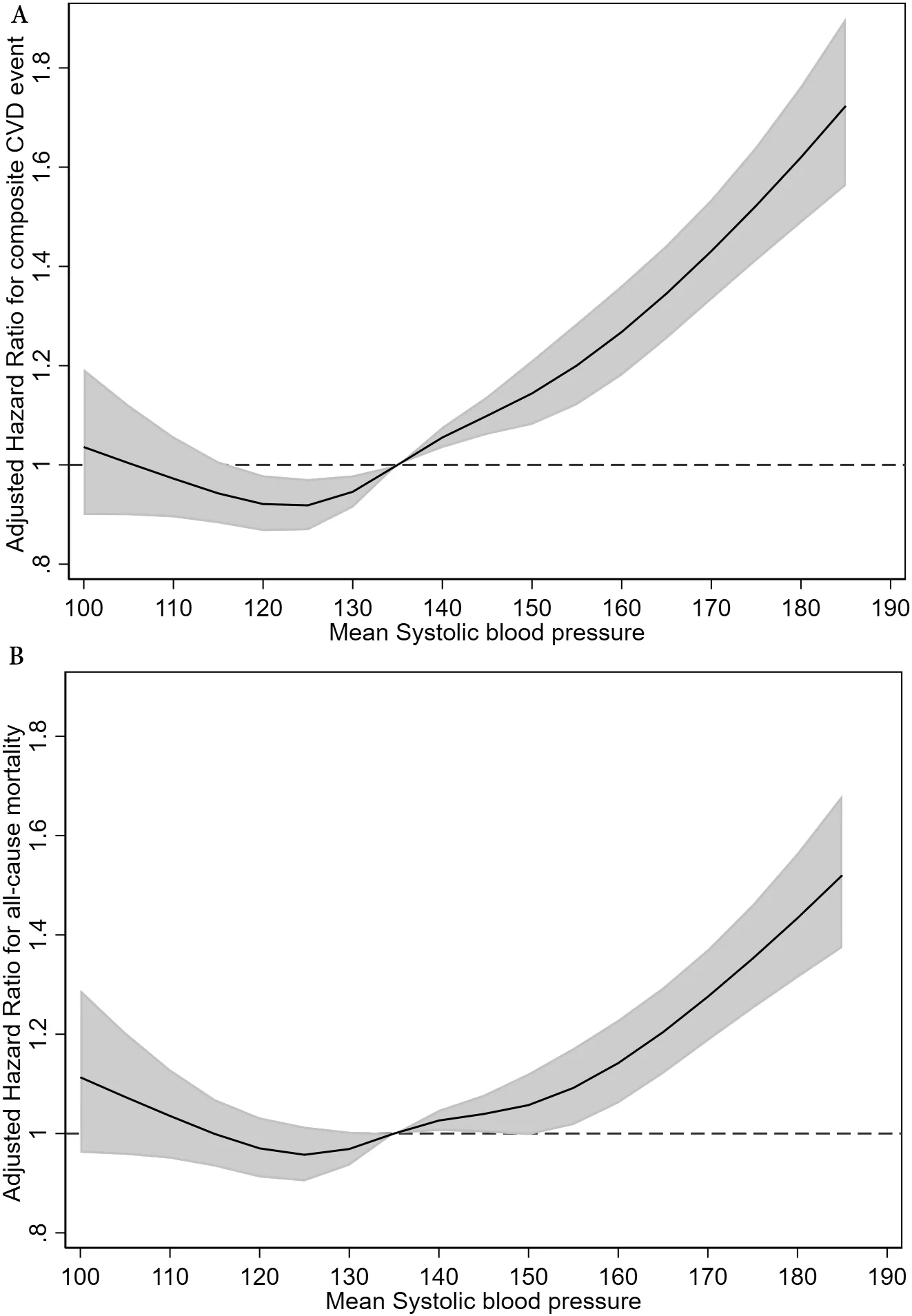
(A) Association between systolic blood pressure and major cardiovascular events. (B) Association between systolic blood pressure and all-cause mortality.

**Table 2:** Incidence rates and adjusted hazard ratios for MACE, all-cause mortality, and the composite of MACE or death according to achieved systolic blood pressure category.

Major adverse cardiovascular events				
Systolic Blood pressure (mmHg)	Number of events/Incidence rate per 100 p-y (95% CI)	Adjusted HR (95% CI)	Adjusted 1-year HR^##^ (95% CI)	Adjusted 3-year HR ^(^95% CI)
100 < 120	986/13.2 (12.4–14.1)	0.96 (0.88–1.04)	1.01 (0.89–1.16)	0.81 (0.72–0.92)
120 < 130	1410/11.4 (10.8–12.0)	0.93 (0.71–0.99)	0.95 (0.84–1.06)	0.91 (0.83–1.01)
130 < 140	1833/11.8 (11.3–12.4)	Reference	Reference	Reference
140 < 150	1818/13.4 (12.8–14.0)	1.06 (0.99–1.13)	1.16 (1.05–1.29)	1.09 (0.99–1.19)
150 < 160	1163/15.0 (14.2–15.9)	1.13 (1.05–1.22)	1.23 (1.08–1.39)	1.10 (0.99–1.23)
≥160	1346/18.5 (17.5–19.5)	1.38 (1.28–1.49)	1.42 (1.26–1.60)	1.38 (1.24–1.55)
All-cause mortality				
Systolic Blood pressure (mmHg)	Number of events/Incidence rate per 100 p-y (95% CI)	Adjusted HR (95% CI)	Adjusted 1-year HR^##^ (95% CI)	Adjusted 3-year HR ^(^95% CI)
100 < 120	887/10.8 (10.1–11.5)	0.97 (0.90–1.04)	1.03 (0.92–1.16)	0.96 (0.86–1.08)
120 < 130	1222/9.1 (8.7–9.7)	0.93 (0.87–0.99)	0.92 (0.83–1.03)	0.97 (0.87–1.07)
130 < 140	1662/9.8 (9.4–10.3)	Reference	Reference	Reference
140 < 150	1580/10.6 (10.1–11.1)	1.02 (0.96–1.08)	0.99 (0.89–1.11)	1.03 (0.93–1.13)
150 < 160	1057/12.0 (11.3–12.7)	1.10 (1.03–1.17)	1.01 (0.90–1.13)	1.12 (1.01–1.24)
≥160	1230/14.4 (13.6–15.2)	1.29 (1.20–1.38)	1.16 (1.04–1.30)	1.21 (1.09–1.34)
Major adverse cardiovascular events + mortality				
	Number of events/Incidence rate per 100 p-y (95% CI)	Adjusted HR (95% CI)	Adjusted 1-year HR^##^ (95% CI)	Adjusted 3-year HR ^(^95% CI)
100 < 120	1254/16.8 (15.9–17.7)	0.98 (0.90–1.06)	1.01 (0.90–1.14)	0.84 (0.76–0.93)
120 < 130	1827/14.8 (14.1–15.5)	0.93 (0.86–0.99)	0.95 (0.86–1.04)	0.92 (0.84–0.99)
130 < 140	2408/15.6 (14.9–16.2)	Reference	Reference	Reference
140 < 150	2338/17.3 (16.6–18.0)	0.97 (0.90–1.04)	1.09 (0.99–1.20)	1.04 (0.97–1.13)
150 < 160	1524/19.8 (18.7–20.7)	1.03 (0.95–1.12)	1.16 (1.04–1.28)	1.09 (0.99–1.19)
≥160	1729/23.7 (22.6–24.9)	1.17 (1.08–1.27)	1.30 (1.17–1.44)	1.29 (1.17–1.41)

Incidence rates are presented per 100 person-years with 95% confidence intervals. Hazard ratios were estimated using multivariable-adjusted flexible parametric proportional hazards models. The 130–<140 mmHg SBP category was used as the reference group. Time-specific HRs represent estimated relative hazards at 1 and 3 years after baseline. MACE was defined as the first occurrence of nonfatal myocardial infarction, coronary heart disease, stroke, heart failure, or cardiovascular death. The composite outcome “MACE or death” was defined as the first occurrence of MACE or death from any cause.

*Models were adjusted for age, sex, eGFR, primary kidney disease, comorbidities, laboratory values, medication use, and calendar year, as specified in the Methods*.

When SBP was analyzed continuously with 125 mmHg as the reference, MACE risk increased progressively at higher SBP levels. At 3 years, the adjusted HRs were 1.08 (95% CI 1.05–1.11) at 135 mmHg, 1.21 (95% CI 1.14–1.29) at 145 mmHg, and 1.56 (95% CI 1.17–2.08) at 165 mmHg (Table 3). We observed no additional benefit or harm with SBPs lower than 120 mmHg relative to 125 mmHg in our main analysis. Cumulative incidence analyses, accounting for competing non-CVD death, supported the primary findings (Fig. 2A, B, and Supplementary Fig. S2) and our composite endpoint including both MACE and all-cause death corroborated the main findings (Table 2). Based on the MACE-specific CIF in male average-aged patients with no comorbidities or medications, who had mean levels of laboratory and clinical values, we observed consistently lower risks over time for all SBP categories <140 mmHg (Fig. 2A).

**Figure 2:**
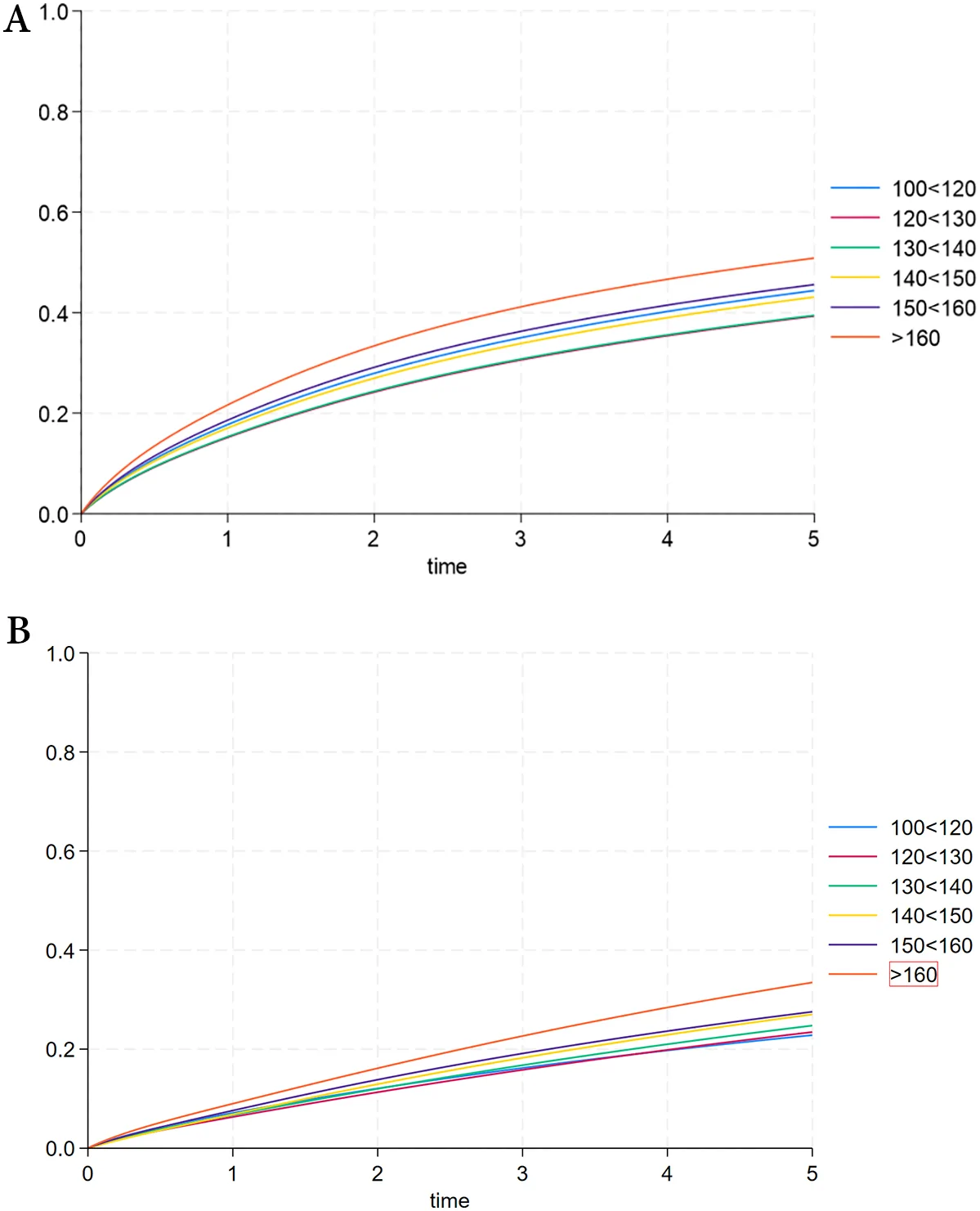
(A) Unadjusted cumulative incidence for the risk of major cardiovascular events according to different systolic blood pressure categories. (B) Adjusted cumulative incidence for the risk of major cardiovascular events according to different systolic blood pressure categories*. * predicted from a flexible parametric model with time-varying blood pressure for male average-aged patients with no comorbidities or medications, who had mean levels of laboratory and clinical values.

**Table 3:** Predicted associations for MACE and All-cause mortality outcomes associated with a reference systolic blood pressure of 125 mmHg in all patients with at least one year of follow-up.

	1-year adjusted HR (95% CI)	3-year adjusted HR (95% CI)^#^
Major cardiovascular event^##^ Blood pressure (mmHg)		
105	1.00 (0.90–1.11)	0.97 (0.87–1.09)
115	0.99 (0.94–1.03)	0.97 (0.93–1.02)
125	Reference	Reference
135	1.06 (1.04–1.09)	1.08 (1.05–1.11)
145	1.17 (1.11–1.24)	1.21 (1.14–1.29)
155	1.31 (1.07–1.61)	1.37 (1.11–1.70)
165	1.47 (1.11–1.94)	1.56 (1.17–2.08)
All-cause mortality		
105	1.07 (0.96–1.19)	1.03 (0.92–1.15)
115	1.02 (0.98–1.07)	1.01 (0.96–1.05)
125	Reference	Reference
135	1.00 (0.98–1.03)	1.02 (1.00–1.05)
145	1.04 (0.98–1.09)	1.07 (1.02–1.13)
155	1.09 (0.88–1.35)	1.15 (0.93–1.43)
165	1.17 (0.88–1.55)	1.26 (0.94–1.68)

Hazard ratios were estimated from multivariable-adjusted flexible parametric proportional hazards models with SBP modelled continuously. An SBP of 125 mmHg was used as the reference value. Estimates are presented at selected SBP values to illustrate the shape of the association. Time-specific HRs represent estimated relative hazards at 1 and 3 years after baseline. Models were adjusted for the covariates specified in the Methods.

*Continuous SBP was modelled using restricted cubic splines*.

### All-cause mortality and cardiovascular death

The association between SBP and all-cause mortality followed a J-shaped curve (Fig. 1B). The risk of death was lower for patients between 120 < 130 mmHg [HR 95% CI 0.93 (0.87—0.99), Table 2]. Patients with SBP >150 mmHg had higher all-cause mortality compared to the reference SBP between 130 < 140, while and risk of cardiovascular death increased for SBP ≥160 mmHg (adjusted HR 1.19; 95% CI 1.03–1.38; Table 4). There was no additional benefit of a SBP lower than 120 mmHg, compared to 125 mmHg (Table 3).

**Table 4:** Incidence rates and adjusted hazard ratios for individual components of MACE according to achieved systolic blood pressure category.

Coronary heart disease				
	Number of events/Incidence rate per 100 p-y (95% CI)	Adjusted HR (95% CI)	Adjusted 1-year HR^##^ (95% CI)	Adjusted 3-year HR ^(^95% CI)
100 < 120	622/7.7 (7.1–8.3)	1.02 (0.89–1.19)	0.84 (0.75–1.16)	1.02 (0.86–1.24)
120 < 130	789/5.8 (5.4–6.2)	0.95 (0.83–1.08)	1.04 (0.86–1.24)	1.02 (0.87–1.19)
130 < 140	987/5.8 (5.4–6.2)	Reference	Reference	Reference
140 < 150	965/6.5 (6.1–6.9)	1.10 (0.97–1.25)	1.31 (1.11–1.55)	1.17 (1.01–1.35)
150 < 160	613/7.0 (6.4–7.5)	1.02 (0.88–1.17)	1.37 (1.13–1.57)	1.33 (1.12–1.57)
≥160	733/8.7 (8.1–9.4)	1.19 (1.03–1.38)	1.40 (1.15–1.70)	1.56 (1.31–1.84)
Myocardial infarction				
	Number of events/Incidence rate per 100 p-y (95% CI)	Adjusted HR (95% CI)	Adjusted 1-year HR^##^ (95% CI)	Adjusted 3-year HR ^(^95% CI)
100 < 120	140/1.6 (1.3–1.9)	0.67 (0.55–0.91)	0.67 (0.50–0.91)	0.66 (0.51–0.85)
120 < 130	275/1.9 (1.7–2.2)	0.91 (0.77–1.07)	0.98 (0.78–1.24)	1.07 (0.88–1.31)
130 < 140	382/2.1 (1.9–2.3)	Reference	Reference	Reference
140 < 150	428/2.7 (2.5–3.0)	1.19 (1.03–1.37)	1.38 (1.12–1.70)	1.23 (1.03–1.47)
150 < 160	249/2.7 (2.4–3.1)	1.13 (0.96–1.33)	1.36 (1.08–1.72)	1.17 (0.95–1.45)
≥160	356/4.0 (3.6–4.5)	1.69 (1.44–1.98)	1.93 (1.55–2.41)	1.80 (1.47–2.19)
Cardiovascular death				
	Number of events/Incidence rate per 100 p-y (95% CI)	Adjusted HR (95% CI)	Adjusted 1-year HR^##^ (95% CI)	Adjusted 3-year HR ^(^95% CI)
100 < 120	308/3.4 (3.1–3.8)	1.03 (0.89–1.19)	1.08 (0.88–1.27)	1.04 (0.86–1.25)
120 < 130	397/2.7 (2.5–3.0)	0.96 (0.83–1.08)	0.96 (0.80–1.15)	1.00 (0.85–1.18)
130 < 140	531/2.9 (2.6–3.1)	Reference	Reference	Reference
140 < 150	568/3.4 (3.2–3.7)	1.10 (0.97–1.08)	1.13 (0.95–1.34)	1.24 (1.07–1.45)
150 < 160	327/3.4 (3.0–3.8)	1.02 (0.88–1.17)	1.02 (0.83–1.24)	1.10 (0.92–1.31)
≥160	396/4.2 (3.8–4.6)	1.19 (1.03–1.38)	1.24 (1.02–1.50)	1.25 (1.05–1.50)
Heart failure				
	Number of events/Incidence rate per 100 p-y (95% CI)	Adjusted HR (95% CI)	Adjusted 1-year HR^##^ (95% CI)	Adjusted 3-year HR ^(^95% CI)
100 < 120	986/13.2 (12.4–14.1)	1.00 (0.90–1.12)	1.11 (0.94–1.32)	0.76 (0.64–0.89)
120 < 130	1410/11.4 (10.8–12.0)	0.95 (0.86–1.04)	0.96 (0.81–1.12)	0.89 (0.78–1.01)
130 < 140	1833/11.8 (11.3–12.4)	Reference	Reference	Reference
140 < 150	1818/13.4 (12.8–14.0)	1.07 (0.97–1.17)	1.17 (1.01–1.34)	1.03 (0.91–1.17)
150 < 160	1163/15.0 (14.2–15.9)	1.08 (0.97–1.20)	1.17 (0.99–1.38)	1.03 (0.89–1.18)
≥160	1346/18.5 (17.5–19.5)	1.35 (1.22–1.50)	1.55 (1.32–1.82)	1.23 (1.06–1.42)
Stroke				
	Number of events/Incidence rate per 100 p-y (95% CI)	Adjusted HR (95% CI)	Adjusted 1-year HR^##^ (95% CI)	Adjusted 3-year HR ^(^95% CI)
100 < 120	176/2.0 (1.7–2.3)	1.00 (0.82–1.21)	0.96 (0.88–1.24)	0.96 (0.88–1.24)
120 < 130	278/2.0 (1.7–2.2)	0.97 (0.82–1.14)	0.93 (0.87–0.99)	0.93 (0.87–0.99)
130 < 140	373/2.1 (1.9–2.3)	Reference	Reference	Reference
140 < 150	348/2.2 (2.0–2.4)	0.95 (0.82–1.11)	1.06 (0.99–1.13)	1.06 (0.99–1.13)
150 < 160	238/2.6 (2.3–2.9)	1.10 (0.93–1.30)	1.13 (1.05–1.22)	1.13 (1.05–1.22)
≥160	308/3.4 (3.1–3.8)	1.43 (1.21–1.69)	1.38 (1.28–1.49)	1.38 (1.28–1.49)

Incidence rates are presented per 100 person-years with 95% confidence intervals. Hazard ratios were estimated using multivariable-adjusted flexible parametric proportional hazards models. The 130–<140 mmHg SBP category was used as the reference group. Time-specific HRs represent estimated relative hazards at 1 and 3 years after baseline. Individual MACE components were analyzed separately and should not be interpreted as mutually exclusive events unless otherwise stated.

### Myocardial infarction, coronary heart disease, heart failure, and stroke

Most individual components of MACE showed similar risk associations as the main analysis, but without statistical significance except for the highest SBP categories. An exception was myocardial infarction (MI), where patients with SBP 100–<120 mmHg had a lower risk than the reference group, and risks increased at higher SBP categories (Table 4; Supplementary Fig. S3). Continuous analyses showed a similar pattern (Table S5). A lower 3-year hazard was also observed for heart failure in the SBP 100–<120 mmHg category (HR 0.76; 95% CI 0.64–0.89; Table 4; Supplementary Fig. S6), whereas no such reduction was seen for coronary heart disease (Table 3; Supplementary Fig. S5). The corresponding associations for MACE including noncardiovascular death, cardiovascular death, and stroke are shown in Supplementary Figs S2, S4, and S7, respectively.

### Subgroup and sensitivity analyses

No significant interactions were found for age (≥65 vs <65 years), sex, diabetes status, comorbidity burden (Charlson comorbidity index > 4), or albuminuria levels (A1/A2 vs A3).

Sensitivity analyses restricted to patients with eGFR <30 ml/min/1.73 m² at index date (*n* = 16 822) and analyses adjusted for albuminuria showed results that were generally consistent with the primary findings (Table S6). These analyses and the baseline characteristics of the broader cohort with eGFR <60 ml/min/1.73 m² (Table S7) showed that this group was slightly older and had more comorbidities compared with the main cohort. We then analyzed all patients with eGFR <60 ml/min/1.73 m² from their first eGFR and SBP measurement regardless of follow-up duration; these individuals had a higher risk of both MACE and all-cause mortality in the SBP category below 120 mmHg (Table S8).

Finally, sensitivity analyses using alternative inclusion time frames, and with KRT as a time-dependent covariate further supported the robustness of the main results, with progressively higher risks of MACE, MACE or death, and all-cause mortality at SBP ≥140 mmHg and the highest risks at SBP ≥160 mmHg, while estimates for SBP 100–120 mmHg remained comparable to the reference category 130–140 mmHg across most analyses (Tables S8–S10).

## DISCUSSION

In this nationwide observational cohort of nephrology-referred patients with CKD, we made four main observations. First, higher SBP was associated with progressively higher cardiovascular risk, with substantially increased MACE rates above 150 mmHg and elevated 1-year risks already at SBP ≥140 mmHg. Second, patients with achieved SBP 120–<130 mmHg had both lower MACE rates and lower all-cause mortality than patients at the standard range of 130–<140 mmHg. Third, compared to a continuous SBP of 125 mmHg, achieved SBP below 120 mmHg did not confer clear additional average benefit. Fourth, although our landmark analyses restricted to patients with stable follow-up did not demonstrate any excess mortality with SBP <120 mmHg, these SBP levels were associated with higher mortality in the broader cohort without this restriction, suggesting that more intensive lowering may be reasonable in patients who tolerate standard levels over time, but potentially hazardous in frail or unstable patients. These associations were consistent across age, sex, diabetes status, comorbidity burden, and albuminuria category.

Although both randomized trials and observational studies consistently show that lower BP is associated with reduced CVD events in patients with mild-to-moderate CKD, evidence specifically supporting more intensive BP lowering in advanced CKD (eGFR <30 ml/min/1.73 m²) is limited [¹⁰, ³²]. These uncertainties are compounded in the broader populations seen in nephrology clinics, elderly patients, those with diabetes, multiple comorbidities, polycystic kidney disease, or severely increased albuminuria [³³], and some studies even raise the possibility of harm with lower SBP targets in such patients, consistent with a J-shaped association [²³, ²⁵, ³³]. Our study therefore addresses a clinically important knowledge gap on the benefits and harms of lower versus higher SBP in patients with predominantly advanced-stage CKD and substantial comorbidity.

In the pre-specified CKD subgroup of SPRINT, the primary composite cardiovascular outcome was not significantly reduced by intensive BP lowering although all-cause mortality was lower [6]. Our findings in nephrology-referred CKD patients are directionally consistent with this signal: patients achieving 120–<130 mmHg had ∼7% lower risk of MACE and of all-cause mortality than those at the standard range of 130–<140 mmHg. Importantly, however, we found no additional overall benefit of achieving an SBP below 120 mmHg relative to 125 mmHg, although our results suggest that some stable patients who maintain SBP 100–<120 mmHg over time may experience lower long-term MACE risk. This pattern corresponds more closely to the ESH/ERA target of <130/80 mmHg (with explicit caution against <120/70 mmHg) [8] than to the universal <120 mmHg target advocated by KDIGO.

In a separate publication from the same nationwide SRR–CKD cohort, we previously examined hard kidney outcomes (CKD progress, chronic dialysis initiation, kidney transplantation, or death) [34]. Here, we focus on MACE and mortality, while still including events after kidney replacement therapy, to complement that kidney–outcome–focused analysis and clarify the cardiovascular implications of achieved SBP in advanced CKD.

In patients with type 2 diabetes and overt nephropathy, the Irbesartan Type II Diabetic Nephropathy Trial suggested benefit at a target of 135/85 mmHg, although effects specific to ARB therapy could not be excluded [35]. The optimal BP target in diabetic CKD remains debated [36] and a meta-analysis by Malhotra *et al*. found reduced mortality with SBP lowering from 148 to 132 mmHg, but no further benefit below 125 mmHg [¹⁰]. A pooled analysis including SPRINT and the AASK trial suggested that intensive control (<130 mmHg) may reduce mortality, particularly after excluding patients with higher eGFR and intensive glycemic targets [37].

A frequent concern when contrasting routine clinical practice with SPRINT is that BP measurement methods differ. Our BP values were obtained as attended clinic readings rather than with SPRINT’s largely unattended protocol, which has been difficult to compare with other trials [³⁸]. However, within SPRINT itself, similar BP levels and cardiovascular risk reductions were observed regardless of whether measurements were attended or unattended [39, 42], and the SPRINT Ambulatory BP Ancillary Study confirmed close agreement between clinic and 24-h ambulatory readings [43]. Thus, while measurement modality differs, the direction and magnitude of benefit from lower SBP appear robust to how BP is measured. Nonetheless, attended clinic readings in routine care remain susceptible to white-coat and masked hypertension, and our findings should be interpreted alongside evidence from unattended office and ambulatory BP measurements.

Our sensitivity analyses provide additional clinical context. When the requirement for 1-year stable follow-up was relaxed, patients with a single SBP <120 mmHg had higher all-cause and cardiovascular mortality. We interpret this group as a more vulnerable subset, whose frailty is unlikely to be fully captured by administrative coding, and importantly, a population typically under-represented in trials such as SPRINT. A plausible interpretation, supported by the contrast between our main landmark analysis, sensitivity analyses and by the time-varying hazard ratios for the lowest SBP group 100–<120 mmHg (where 3-year risks were lower than in the 130–<140 mmHg group despite neutral or slightly higher early hazards), is that stricter SBP control below 120 mmHg may be appropriate in patients who first demonstrate tolerance to lower levels (120–<130 mmHg) over an initial observation period, whereas more aggressive lowering in frail or unstable patients may be hazardous.

Our study has several notable strengths. It is the largest cohort study to date of patients with advanced-stage CKD (mean eGFR ∼23 ml/min/1.73 m²), based on a nationally representative sample with minimal loss to follow-up enabled by comprehensive healthcare data linkages. The cohort was well-characterized, with detailed information on laboratory values, comorbidities, and prescribed medications, and the intensive and standard BP groups remained separated throughout follow-up despite the use of a one-year baseline window. Importantly, our cohort reflects real-world nephrology practice, including a substantial proportion of older patients and patients with diabetes, so by mimicking key features of the SPRINT design in this broader population, where only about 14% would have met SPRINT eligibility criteria, we extend the evidence base to a more clinically relevant CKD group seen in secondary nephrology care.

Several limitations should be acknowledged. As an observational design, the study cannot establish causality, and the bidirectional relationship between BP and CKD complicates interpretation. The cohort, derived from specialist nephrology care, includes more advanced kidney disease than typically seen in primary care, which may limit generalizability. The observed rate ratios should be read as describing underlying disease and treatment strategies used to achieve a given BP, recognizing that only randomized trials can definitively determine optimal targets. Furthermore, estimates reflect cause-specific rates, although predicted cumulative incidence curves accounting for competing risks were consistent with the main findings. BP measurements were not standardized at every visit, but reflect real-world practice and patient adherence, and any resulting bias is likely nondifferential. We do not focus on visit-to-visit BP variability, which has independently been linked to adverse outcomes in CKD [44]. Although no significant interaction was found for albuminuria, the relatively small number of patients with normo-albuminuria and the substantial proportion with missing albuminuria measurements may have limited power to detect such effects, so residual confounding cannot be excluded. Finally, our main analyses do not inform management of patients with sustained SBP <100 mmHg; this clinically distinct group, in whom low BP often reflects advanced comorbidity (e.g. heart failure) or excessive antihypertensive treatment, requires careful individualization, and our results should not be extrapolated to suggest that deliberately targeting SBP <100 mmHg is safe or beneficial.

In conclusion, this large observational study of nephrology-referred patients with CKD demonstrates that an achieved SBP near 125 mmHg is associated with the lowest incidence of cardiovascular events, while SBP ≥140 mmHg is consistently associated with higher risk and SBP ≥160 mmHg with the greatest risk. We did not observe clear additional average benefit from achieving SBP below 120 mmHg, but stable patients who tolerated SBP 100–<120 mmHg over time had lower long-term MACE risk than those at 130–<140 mmHg, underlining the importance of individualized targets.

## Supplementary Material

sfag296_Supplemental_File

## Data Availability

The data underlying this article cannot be shared publicly due to the privacy of individuals that participated in the study. The data may be shared on reasonable request for academic research collaborations that fulfill GDPR as well as national and institutional ethics regulations and standards by contacting Dr Marie Evans (marie.evans@ki.se).

## References

[1] Ortiz A, Lees JS, Torra R et al. The updated global burden of chronic kidney disease: one death every 20 seconds. Nephrol Dial Transplant. 2026. 10.1093/ndt/gfag040 PMC13624808 41739048

[2] Ninomiya T, Perkovic V, Turnbull F et al. Blood pressure lowering and major cardiovascular events in people with and without chronic kidney disease: meta-analysis of randomised controlled trials. BMJ. 2013;347:f5680. 10.1136/bmj.f5680 PMC3789583 24092942

[3] Bundy JD, Li C, Stuchlik P et al. Systolic blood pressure reduction and risk of cardiovascular disease and mortality: a systematic review and network meta-analysis. JAMA Cardiol. 2017;2:775–81. 10.1001/jamacardio.2017.1421 PMC5710614 28564682

[4] Wright JT, Williamson JD, Whelton PK et al. A randomized trial of intensive versus standard blood-pressure control. N Engl J Med. 2015;373:2103–16. 10.1056/NEJMoa1511939 PMC4689591 26551272

[5] KDIGO 2021. Clinical Practice Guideline for the management of blood pressure in chronic kidney disease. Kidney Int. 2021;99:S1–S87. 10.1016/j.kint.2020.11.003 33637192

[6] Cheung AK, Rahman M, Reboussin DM et al. Effects of intensive BP control in CKD. J Am Soc Nephrol. 2017;28:2812–23. 10.1681/ASN.2017020148 PMC5576945 28642330

[7] Dasgupta I, Zoccali C. Is the KDIGO Systolic Blood Pressure Target <120 mm Hg for Chronic Kidney Disease Appropriate in Routine Clinical Practice?. Hypertension. 2022;79:4–11. 10.1161/HYPERTENSIONAHA.121.18434 PMC8654101 34784720

[8] Mancia G, Kreutz R, Brunström M et al. ESH Guidelines for the management of arterial hypertension The Task Force for the management of arterial hypertension of the European Society of Hypertension: endorsed by the International Society of Hypertension (ISH) and the European Renal Association (ERA). J Hypertens. 2023;41:1874–2071. 10.1097/HJH.0000000000003480 37345492

[9] Tada K, Fujiwara A, Sugano N et al. Evaluating blood pressure targets in chronic kidney disease: a systematic review and meta-analysis. Hypertens Res. 2025;48:2358–67. 10.1038/s41440-025-02262-4 40542174

[10] Malhotra R, Nguyen HA, Benavente O et al. Association between more intensive vs less intensive blood pressure lowering and risk of mortality in chronic kidney disease stages 3 to 5: a systematic review and meta-analysis. JAMA Intern Med. 2017;177:1498–505. 10.1001/jamainternmed.2017.4377 PMC5704908 28873137

[11] Turner R, Holman R, Stratton I et al. Tight blood pressure control and risk of macrovascular and microvascular complications in type 2 diabetes: UKPDS 38. UK Prospective Diabetes Study Group. BMJ. 1998;317:703–13. 10.1136/bmj.317.7160.703 PMC28659 9732337

[12] Wright JT Jr et al. Effect of blood pressure lowering and antihypertensive drug class on progression of hypertensive kidney disease: results from the AASK trial. JAMA. 2002;288:2421–31. 10.1001/jama.288.19.2421 12435255

[13] Levey AS, Greene T, Beck GJ et al. Dietary protein restriction and the progression of chronic renal disease: what have all of the results of the MDRD study shown? Modification of Diet in Renal Disease Study group. J Am Soc Nephrol. 1999;10:2426–39. 10.1681/ASN.V10112426 10541304

[14] Ruggenenti P, Perna A, Loriga G et al. Blood-pressure control for renoprotection in patients with non-diabetic chronic renal disease (REIN-2): multicentre, randomised controlled trial. Lancet. 2005;365:939–46. 10.1016/S0140-6736(05)71082-5 15766995

[15] Tonelli M, Muntner P, Lloyd A et al. Using proteinuria and estimated glomerular filtration rate to classify risk in patients with chronic kidney disease: a cohort study. Ann Intern Med. 2011;154:12–21. 10.7326/0003-4819-154-1-201101040-00003 21200034

[16] Koye DN, Magliano DJ, Reid CM et al. Risk of progression of nonalbuminuric CKD to end-stage kidney disease in people with diabetes: the CRIC (Chronic Renal Insufficiency Cohort) Study. Am J Kidney Dis. 2018;72:653–61. 10.1053/j.ajkd.2018.02.364 29784612

[17] Brown WW, Peters RM, Ohmit SE et al. Early detection of kidney disease in community settings: the Kidney Early Evaluation Program (KEEP). Am J Kidney Dis. 2003;42:22–35. 10.1016/S0272-6386(03)00405-0 12830453

[18] Young JH, Klag MJ, Muntner P et al. Blood pressure and decline in kidney function: findings from the Systolic Hypertension in the Elderly Program (SHEP). J Am Soc Nephro. 2002;13:2776–82. 10.1097/01.ASN.0000031805.09178.37 12397049

[19] Smink PA, Lambers Heerspink HJ, Gansevoort RT et al. Albuminuria, estimated GFR, traditional risk factors, and incident cardiovascular disease: the PREVEND (Prevention of Renal and Vascular Endstage Disease) study. Am J Kidney Dis. 2012;60:804–11. 10.1053/j.ajkd.2012.06.017 22835901

[20] Romundstad S, Holmen J, Kvenild K et al. Microalbuminuria and all-cause mortality in 2,089 apparently healthy individuals: a 4.4-year follow-up study. The Nord-Trøndelag Health Study (HUNT), Norway. Am J Kidney Dis. 2003;42:466–73. 10.1016/S0272-6386(03)00742-X 12955674

[21] Kitamura H, Tanaka S, Hiyamuta H et al. Associations of causes of chronic kidney disease with disease progression and mortality: insights from the Fukuoka Kidney Disease Registry Study. Am J Nephrol. 2025;56:366–76. 10.1159/000543201 39778545

[22] Kim HW, Park JT, Joo YS et al. Systolic blood pressure and chronic kidney disease progression in patients with primary glomerular disease. J Nephrol. 2021;34:1057–67. 10.1007/s40620-020-00930-x 33555575

[23] Ku E, McCulloch CE, Vittinghoff E et al. Use of antihypertensive agents and association with risk of adverse outcomes in chronic kidney disease: focus on angiotensin-converting enzyme inhibitors and angiotensin receptor blockers. J Am Heart Assoc. 2018;7:e009992. 10.1161/JAHA.118.009992 PMC6404880 30371331

[24] Flint AC, Conell C, Ren X et al. Effect of systolic and diastolic blood pressure on cardiovascular outcomes. N Engl J Med. 2019;381:243–51. 10.1056/NEJMoa1803180 31314968

[25] Zhuo M, Yang D, Goldfarb-Rumyantzev A et al. The association of SBP with mortality in patients with stage 1-4 chronic kidney disease. J Hypertens. 2021;39:2250–7. 10.1097/HJH.0000000000002927 PMC8500924 34232158

[26] Registry SR Annual report 2025. Svenskt Njurregister (SNR). Årsrapport 2025. Stockholm: Svenskt Njurregister; 2025. 2025. Available from: https://www.medscinet.net/snr/rapporterdocs/%C3%85rsrapport%202025.pdf (March 2026, date last accessed).

[27] Ludvigsson JF, Andersson E, Ekbom A et al. External review and validation of the Swedish national inpatient register. BMC Public Health. 2011;11:450. 10.1186/1471-2458-11-450 PMC3142234 21658213

[28] Wettermark B, Zoëga H, Furu K et al. The Nordic prescription databases as a resource for pharmacoepidemiological research—a literature review. Pharmacoepidemiol Drug. 2013;22:691–9. 10.1002/pds.3457 23703712

[29] Fine JP, Gray RJ. A proportional hazards model for the subdistribution of a competing risk. J Am Statist Assoc. 1999;94:496–509. 10.2307/2670170

[30] Merlin CM. A unified modelling framework for data analysis and methods development in Stata. StataJ. 2020;20:763–84.

[31] Stata Statistical Software: Release 18. StataCorp LLC; 2023.

[32] Xie Y, Bowe B, Mokdad AH et al. Analysis of the Global Burden of Disease study highlights the global, regional, and national trends of chronic kidney disease epidemiology from 1990 to 2016. Kidney Int. 2018;94:567–81. 10.1016/j.kint.2018.04.011 30078514

[33] Messerli FH, Panjrath GS. The J-curve between blood pressure and coronary artery disease or essential hypertension: exactly how essential?. J Am Coll Cardiol. 2009;54:1827–34. 10.1016/j.jacc.2009.05.073 19892233

[34] Al-Sodany E, Chesnaye NC, Heimbürger O et al. Blood pressure and kidney outcomes in patients with severely decreased glomerular filtration rate: a nationwide observational cohort study. J Hypertens. 2022;40:1487–98. 10.1097/HJH.0000000000003168 PMC9415216 35730420

[35] Berl T, Hunsicker LG, Lewis JB et al. Cardiovascular outcomes in the Irbesartan Diabetic Nephropathy Trial of patients with type 2 diabetes and overt nephropathy. Ann Intern Med. 2003;138:542–9. 10.7326/0003-4819-138-7-200304010-00010 12667024

[36] Bakris GL, Williams M, Dworkin L et al. Preserving renal function in adults with hypertension and diabetes: a consensus approach. National Kidney Foundation Hypertension and Diabetes Executive Committees Working Group. Am J Kidney Dis. 2000;36:646–61. 10.1053/ajkd.2000.16225 10977801

[37] Aggarwal R, Petrie B, Bala W et al. Mortality outcomes with intensive blood pressure targets in chronic kidney disease patients. Hypertension. 2019;73:1275–82. 10.1161/HYPERTENSIONAHA.119.12697 31067189

[38] Drawz PE, Agarwal A, Dwyer JP et al. Concordance between blood pressure in the systolic blood pressure intervention trial and in routine clinical practice. JAMA Intern Med. 2020;180:1655–63. 10.1001/jamainternmed.2020.5028 PMC7551238 33044494

[39] Kjeldsen SE, Mancia G. Unobserved automated office blood pressure measurement in the Systolic Blood Pressure Intervention Trial (SPRINT): systolic blood pressure treatment target remains below 140 mmHg. Eur Heart J Cardiovasc Pharmacother. 2016;2:79–80. 10.1093/ehjcvp/pvw002 27533517

[40] Agarwal R. Implications of blood pressure measurement technique for implementation of Systolic Blood Pressure Intervention Trial (SPRINT). J Am Heart Assoc. 2017;6:e004536. 10.1161/jaha.116.004536 PMC5523757 28159816

[41] Drawz PE, Pajewski NM, Bates JT et al. Effect of intensive versus standard clinic-based Hypertension Management on Ambulatory Blood Pressure: results From the SPRINT (Systolic Blood Pressure Intervention Trial) Ambulatory Blood Pressure Study. Hypertension. 2017;69:42–50. 10.1161/HYPERTENSIONAHA.116.08076 PMC5145774 27849563

[42] Johnson KC, Whelton PK, Cushman WC et al. Blood pressure measurement in SPRINT (Systolic Blood Pressure Intervention Trial). Hypertension. 2018;71:848–57. 10.1161/HYPERTENSIONAHA.117.10479 PMC5967644 29531173

[43] Ghazi L, Cohen LP, Muntner P et al. Effects of intensive versus standard office-based hypertension treatment strategy on white-coat effect and masked uncontrolled hypertension: from the SPRINT ABPM Ancillary Study. Hypertension. 2020;76:1090–6. 10.1161/HYPERTENSIONAHA.120.15300 PMC7484232 32829666

[44] Mallamaci F, Minutolo R, Leonardis D et al. Long-term visit-to-visit office blood pressure variability increases the risk of adverse cardiovascular outcomes in patients with chronic kidney disease. Kidney Int. 2013;84:381–9. 10.1038/ki.2013.132 23615498

