# Supplementary material for: Achieved systolic blood pressure and cardiovascular outcomes in advanced chronic kidney disease
Source: Clin Kidney J. 2026 Aug 31;19(10):sfag296. doi: 10.1093/ckj/sfag296 (PMC13655453; doi:10.1093/ckj/sfag296)
Supplement: Supplementary file 1 [file sfag296_Supplemental_File.docx]

**Supplementary Appendix**

**Achieved systolic blood pressure in severe chronic kidney disease patients and cardiovascular outcomes: a nationwide cohort study**

Al-Sodany E, Szummer K, Österman J, Dietrich CE, Carrero J-J, Heimburger O, Bárány P, Evans M

**Content:**

General preparations at nephrology clinic for blood pressure measurement and patient’s instruction.

**Tables:**

**Table S1**. Definitions of primary and secondary outcomes (MACE and its components, all‑cause mortality, composite of MACE or death).

**Table S2**. ICD‑10 codes used to classify comorbidities.

**Table S3.** ATC codes used to classify medications.

**Table S4.** Baseline characteristics in patients included in our study cohort (n=23,317) and SPRINT participants with CKD (n=2,646).

**Table S5.** Continuous associations (restricted spline models) for individual MACE components (including myocardial infarction, coronary heart disease, heart failure, stroke) and other outcomes, with 125 mmHg as SBP reference.

**Table S6.** Sensitivity analyses restricted to eGFR <30 ml/min/1.73 m² and analyses adjusted for albuminuria (cause‑specific HRs by SBP category).

**Table S7.** Comparison of characteristics between participants in the main analysis (n=23,317), those excluded from analyses (N=4,812), and those excluded due to SBP <100 mmHg (N=244).

**Table S8.** Outcomes (MACE, all‑cause mortality, MACE or death) in the broader cohort with eGFR <60 ml/min/1.73 m² using first SBP, including those with SBP <100 mmHg and early KRT/death (cause‑specific HRs by SBP category).

**Table S9.** Sensitivity analyses using alternative inclusion time frames (e.g. 6‑ and 12‑month landmarks), showing HRs for MACE, MACE or death, and all‑cause mortality by SBP category.

**Table S10**. Sensitivity analysis with kidney replacement therapy as time-dependent covariate

**Figures:**

**Supplementary Figure S1.** Description of study methods for index visit and follow‑up timeline.

**Supplementary Figure S2**. Association between systolic blood pressure and MACE including non‑cardiovascular death.

**Supplementary Figure S3.** Association between systolic blood pressure and myocardial infarction.

**Supplementary Figure S4.** Association between systolic blood pressure and cardiovascular death.

**Supplementary Figure S5.** Association between systolic blood pressure and coronary arterial disease events.

**Supplementary Figure S6.** Association between systolic blood pressure and heart failure.

**Supplementary Figure S7.** Association between systolic blood pressure and stroke.

**General preparations at nephrology clinics in Sweden for blood pressure measurement and patient’s instruction**

- No talking or use of smartphone during the procedure.
- No exercise, nicotine, or caffeine for at least 15 minutes prior to measurement.
- Remove clothing covering location of cuff.
- Seated comfortably with legs uncrossed and back and arm supported for at least 5 minutes prior to measurement.
- Verify cuff size is correct.

**Additional information regarding methods for Statistical analyses**

Confounders for the adjusted main outcome models: age (non-linear effect), sex, history of myocardial infarction, peripheral arterial disease, atrial fibrillation, congestive heart failure, plasma albumin, phosphate, eGFR (non-linear effect), diastolic blood pressure (non-linear effect) and treatment with angiotensin converting enzyme inhibitors/angiotensin receptor blockers (ACEi/ARB), betablockers, and diuretics.

Cumulative incidence function was predicted for patients with the mean and reference value on all included variables: male, age 69 years, presence of none of the co-morbid conditions included in the model, plasma albumin 37 g/L, phosphate 1,26, eGFR 24 ml/min/1.73m^2^, diastolic BP 77 mmHg. Figure 3 is based upon this prediction.

**Table S1 Definition of study outcomes**

| **Outcome** | **Definition** | **ICD-codes** |
| --- | --- | --- |
| All-cause mortality | Death from any cause during the observational period | n/a  Collected from SRR |
| MACE. Composite endpoint. | Non-fatal myocardial infarction | I210-I21.9, I22.0-I22.9  I23.0-I23.8 |
|  | Coronary heart disease/angina | I20.0-I20.9, I25.0-I25.9 |
|  | Congestive heart failure, non-fatal. | I09.9, I11.0, I13.0, I13.2, I42.0, I42.5-I42.9, I43.0-I43.9, I50.0-I50.9, P29.0 |
|  | Non-fatal Stroke | H34.1, I60.0-I60.9, I61.0-I61.9, I63.0-I63.9, I64.0-I64.9, I69.0-I69.9 |
|  | Cardiovascular death | Any cardiovascular death cause |
| *MACE (Major adverse cardiovascular event).*  *SRR (Swedish Renal Registry)*  *Outcomes were ascertained through linkage with national health registers using ICD-10 codes and cause-of-death information. MACE was defined as a composite of non-fatal myocardial infarction, coronary heart disease, heart failure, non-fatal stroke, or cardiovascular death. For composite outcomes, the first qualifying event during follow-up was used.* | | |

**Table S2** ICD-10 codes used to define comorbidities

| **Diagnosis** | **ICD-codes** |
| --- | --- |
| Coronary heart disease | I21-I23, I25 |
| Diabetes mellitus | E10-E14 |
| Heart failure | I099, I110, I130, I132, I420, I425-I429, I430-I439, I500-I509, P290 |
| Peripheral arterial disease | I700, I710-I719, I731, I738, I739, I771, I790, I792, K551, K558, K559, Z958, Z959 |
| Cerebrovascular disease | G450-G459, G460-G469, H340, I600-I609, I610-I619, I620-I629 I630-I639, I640-I649, I650-I659, I660-I669, I670-I679, I680-I689, I690-I699 |
| Atrial fibrillation | I480/I489 |
| Hypertension | I100-I109, I110-I119, I120-I129, I130-I139, I150-I159 |
| Cancer | C0-C9, D0-D4 |
| *ICD (the International Classification of Diseases)*  *Comorbidities were defined using ICD-10 diagnosis codes recorded before baseline. ICD-10, International Classification of Diseases, 10th Revision.*  *Definitions were based on inpatient and/or specialist outpatient diagnoses available through national register linkage.* | |

**Table S3** ATC codes used to classify medications

| **Medication** | **ATC-codes** |
| --- | --- |
| ACEi/ARBs | C09A, C09B, C09C, C09D |
| Betablockers | C07 |
| CCB | C08 |
| Diuretics | C03, C02L |
| Statin | C10 |
| Insulin | A10A |
| Vitamin K antagonists (warfarin) | B01AA |
| ACEi angiotensin converting enzyme inhibitors. ARB angiotensin 2 receptor blockers. ATC (Anatomical Therapeutic Chemical classification system). CCB (Calcium Channel Blockers). | |
| *Medication use was defined from registered prescriptions or medication records available at baseline, using Anatomical Therapeutic Chemical classification codes, ATC.* | |

**Table S4** Baseline characteristics in patients included in our study cohort (n=23,317) and of the SPRINT participants with CKD (n=2,646).

| **Clinical characteristics** | **Study cohort**  **(n=****23,317)** | **SPRINT participants with CKD**  **(n=2,646)** |
| --- | --- | --- |
| Age (years) mean (SD) | 69.3 (13.6) | 71.9 (9.3) |
| Women (%) | 8,513 (36.5) | 1058 (40.0) |
| **Primary kidney disease (%)** |  |  |
| Polycystic kidney disease and other hereditary | 1,091 (4.7) | 0 |
| Diabetic nephropathy | 4,824 (20.7) | 0 |
| Glomerulonephritis | 2,141 (9.2) | 0 |
| Hypertensive kidney disease | 5,614 (24.1) | n/a |
| Other specified renal diseases | 4,818 (20.7) | n/a |
| Tubulointerstitial disease | 689 (3.0) | n/a |
| Renovascular disease | 174 (0.75) | n/a |
| Unknown | 3,956 (16.8) | n/a |
| **Comorbidity (%)** |  |  |
| Diabetes mellitus | 9,280 (39.9) | 0 |
| Ischemic heart disease | 8,759 (37.7) | n/a |
| Chronic heart failure | 5,297 (22.8) | n/a |
| Cerebrovascular disease | 3,969 (17.1) | n/a |
| Peripheral vascular disease | 2,169 (9.3) | n/a |
| Cancer diagnosis | 8,596 (37.0) | n/a |
| Atrial fibrillation | 4,036 (17.4) | n/a |
| **Clinical data**  mean (SD) |  |  |
| eGFR (ml/min/1.73m^2^) | 25.7 (10.2) | 47.9 (9.5) |
| Systolic BP (mmHg) | 139.2 (18.1) | 139.2 (16.1) |
| Diastolic BP (mmHg) | 77.0 (10.2) | 74.9 (12.2) |
| BMI (kg/m2) | 28.2 (5.7) | 29.4 (5.8) |
| **Routine laboratory data** |  |  |
| U-albumin/ creatinine ratio (mg/mmol) median (IQR) | 29.0 (5.3–125.5) | 1.5 (0.72–4.9) |
| **Current medication no. (%)** |  |  |
| Diuretics | 15,223 (65.3) | Intensive 895 (67.3)  Standard 613 (46.6) |
| ACEi/ARB | 16,241 (69.7) | Intensive 953 (71.7)  Standard 750 (57.0) |
| Betablockares | 14,618 (62.7) | Intensive 694 (52.2)  Standard 555 (42.2) |
| Calcium blockares | 13,372 (57.4) | Intensive 810 (60.9)  Standard 491 (37.3) |
| Statins | 12,772 (54.8) | 1354 (51.5) |
| Antiplatelet therapy | 9638 (41.3) | 1482 (56.1) |
| *N Number. eGFR estimated glomerular filtration rate. BP blood pressure. ACEi angiotensin converting enzyme inhibitors. ARB angiotensin 2 receptor blockers. IQR interquartile range. SD standard deviation. BMI body mass index.*  *Values are presented as n (%), mean (standard deviation), or median (interquartile range), as appropriate. The comparison is descriptive and intended to illustrate differences in case mix, kidney disease severity, albuminuria, comorbidity, and medication use between the present real-world CKD cohort and CKD participants in SPRINT. No formal statistical testing was performed.* | | |

**Table S5** Predicted adjusted hazard ratios for individual MACE components at selected SBP values using 125 mmHg as reference

|  | **1-year adjusted HR (95% CI)** | **3-year adjusted HR (95% CI) ^#^** |
| --- | --- | --- |
| **Myocardial infarction** |  |  |
| 105 | 0.61 (0.46-0.81) | 0.65 (0.50-0.84) |
| 115 | 0.79 (0.70–0.89) | 0.81 (0.73–0.91) |
| 125 | Reference | Reference |
| 135 | 1.21 (1.13-1.30) | 1.18 (1.12-1.24) |
| 145 | 1.42 (1.24-1.63) | 1.25 (1.12-1.41) |
| 155 | 1.67 (0.98–2.87) | 1.53 (0.91–2.59) |
| 165 | 1.99 (0.97-4.06) | 1.77 (0.88–3.56) |
| **Cardiovascular death** |  |  |
| 105 | 1.09 (0.91-1.31) | 1.08 (0.90-1.29) |
| 115 | 1.03 (0.96-1.11) | 1.02 (0.95-1.10) |
| 125 | Reference | Reference |
| 135 | 1.02 (0.98-1.06) | 1.03 (0.99-1.07) |
| 145 | 1.08 (0.99-1.18) | 1.09 (1.01-1.19) |
| 155 | 1.16 (0.81–1.67) | 1.18 (0.82–1.70) |
| 165 | 1.25 (0.77–2.02) | 1.28 (0.79-2.07) |
| **Congestive heart failure** |  |  |
| 105 | 0.99 (0.86-1.14) | 0.98 (0.84- 1.15) |
| 115 | 0.98 (0.93-1.04) | 0.98 (0.92-1.05) |
| 125 | Reference | Reference |
| 135 | 1.05 (1.02-1.09) | 1.06 (1.01-1.11) |
| 145 | 1.15 (1.07-1.24) | 1.16 (1.06-1.27) |
| 155 | 1.27 (0.98–1.66) | 1.29 (0.97–1.72) |
| 165 | 1.43 (1.00–2.02) | 1.46 (1.00-2.13) |
| **Stroke** |  |  |
| 105 | 1.12 (0.88-1.43) | 1.09 (0.86-1.39) |
| 115 | 1.04 (0.94-1.15) | 1.03 (0.93-1.14) |
| 125 | Reference | Reference |
| 135 | 1.02 (0.96-1.07) | 1.03 (0.98-1.09) |
| 145 | 1.09 (0.97-1.22) | 1.12 (1.01-1.25) |
| 155 | 1.20 (0.94–1.93) | 1.25 (0.77–2.02) |
| 165 | 1.34 (0.71-2.53) | 1.42 (0.75–2.70) |
| *Hazard ratios were estimated from multivariable-adjusted flexible parametric proportional hazards models with SBP modelled continuously. An SBP of 125 mmHg was used as the reference value. Estimates are presented at selected SBP values to illustrate the shape of the association for individual MACE components. Time-specific HRs represent estimated relative hazards at 1 and 3 years after baseline. Models were adjusted for age at baseline (restricted cubic spline), sex, diabetes, history of myocardial infarction, peripheral arterial disease, atrial fibrillation, heart failure, plasma albumin, phosphate, eGFR, diastolic blood pressure (restricted cubic spline), current use of diuretics, ACEi/ARB, betablockers*  *Continuous SBP was modelled using restricted cubic splines.* | | |

**Table S6** Subgroup and albuminuria-adjusted analyses according to achieved systolic blood pressure category

| **Major adverse cardiovascular events (MACE)**  Systolic Blood pressure (mmHg) | **Adjusted HR (95% CI)**  **Restricted to patients with eGFR<30 ml/min/1.73m^1^**  **N=16,822** | **Adjusted HR (95% CI)**  **Albuminuria adjusted in all***  **Mean SBP**  **N=23,317** | **Adjusted HR (95% CI)**  **Restricted to patients with albuminuria measurements****  **Mean SBP**  **N=12,649** |
| --- | --- | --- | --- |
| 100<120 | 0.98 (0.89 – 1.08) | 0.98 (0.90—1.07) | 1.12 (0.97-1.29) |
| 120<130 | 0.94 (0.86 – 1.02) | 0.94 (0.88—1.01) | 0.96 (0.84—1.08) |
| 130<140 | Reference | Reference | Reference |
| 140<150 | 1.08 (0.99 – 1.16) | 1.05 (0.98 – 1.25) | 1.10 (0.98—1.24) |
| 150<160 | 1.15 (1.06 – 1.25) | 1.11 (1.03 – 1.20) | 1.14 (1.00—1.40) |
| ≥160 | 1.40 (1.29 – 1.53) | 1.34 (1.24 – 1.45) | 1.25 (1.09—1.46) |
| **All-cause mortality**  Systolic Blood pressure (mmHg) | **Adjusted HR (95% CI)**  **Restricted to patients with eGFR<30 ml/min/1.73m^1^**  **N=16,822** | **Adjusted HR (95% CI)**  **Albuminuria adjusted in all**  **Mean SBP**  **N=23,317** | **Adjusted HR (95% CI)**  **Restricted to patients with albuminuria measurements**  **Mean SBP**  **N=12,649** |
| 100<120 | 0.97 (0.88 – 1.07) | 1.01 (0.93 – 1.11) | 1.03 (0.88—1.21) |
| 120<130 | 0.94 (0.87 – 1.02) | 0.94 (0.87 – 1.01) | 0.90 (0.78—1.03) |
| 130<140 | Reference | Reference | Reference |
| 140<150 | 0.98 (0.91 – 1.06) | 0.96 (0.89—1.03) | 0.89 (0.78—1.02) |
| 150<160 | 1.00 (0.92 – 1.10) | 1.02 (0.94—1.10) | 0.92 (0.79—1.06) |
| ≥160 | 1.17 (1.07 – 1.28) | 1.14 (1.05—1.24) | 0.96 (0.82—1.12) |
| **Cardiovascular death**  Systolic Blood pressure (mmHg) | **Adjusted HR (95% CI)**  **eGFR<30**  **Mean SBP first year**  **N=16,822** | **Adjusted HR (95% CI)**  **Albuminuria adjusted in all**  **Mean SBP**  **N=23,317** | **Adjusted HR^#^ (95% CI)**  **Restricted to patients with albuminuria measurements**  **Mean SBP**  **N=12,649** |
| 100<120 | 1.20 (1.04 – 1.37) | 1.06 (0.91—1.23) | 1.07 (0.81—1.40) |
| 120<130 | 1.03 (0.89 – 1.18) | 0.95 (0.83—1.09) | 0.80 (0.60—1.00) |
| 130<140 | Reference | Reference | Reference |
| 140<150 | 1.16 (1.02 – 1.32) | 1.10 (0.97—1.24) | 1.06 (0.85-1.32) |
| 150<160 | 1.09 (0.94 – 1.27) | 1.02 (0.88—1.18) | 0.82 (0.63—1.08) |
| ≥160 | 1.25 (1.10 – 1.42) | 1.18 (1.02—1.36) | 0.91 (0.69—1.20) |
| **Myocardial infarction**  Systolic Blood pressure (mmHg) | **Adjusted HR (95% CI)**  **eGFR<30**  **Mean SBP first year**  **N=16,822** | **Adjusted HR (95% CI)**  **Albuminuria adjusted in all**  **Mean SBP**  **N=23,317** | **Adjusted HR^#^ (95% CI)**  **Restricted to patients with albuminuria measurements**  **Mean SBP**  **N=12,649** |
| 100<120 | 0.90 (0.70 – 1.15) | 0.69 (0.56—0.85) | 0.61 (0.42—0.89) |
| 120<130 | 0.88 (0.71 – 1.09) | 0.92 (0.78—1.08) | 0.75 (0.56—0.99) |
| 130<140 | Reference | Reference | Reference |
| 140<150 | 1.28 (1.07 – 1.53) | 1.18 (1.02—1.36) | 1.06 (0.83—1.35) |
| 150<160 | 1.23 (1.00 – 1.51) | 1.11 (0.94—1.31) | 1.04 (0.78—1.39) |
| ≥160 | 1.77 (1.48 – 2.13) | 1.64 (1.40—1.93) | 1.31 (0.98—1.74) |
| **Coronary heart disease**  Systolic Blood pressure (mmHg) | **Adjusted HR (95% CI)**  **eGFR<30**  **Mean SBP first year**  **N=16,822** | **Adjusted HR (95% CI)**  **Albuminuria adjusted in all**  **Mean SBP**  **N=23,317** | **Adjusted HR^#^ (95% CI)**  **Restricted to patients with albuminuria measurements**  **Mean SBP**  **N=12,649** |
| 100<120 | 0.88 (0.75 – 1.03) | 0.88 (0.76—1.00) | 0.88 (0.70—1.12) |
| 120<130 | 1.03 (0.91 – 1.18) | 1.00 (0.89—1.12) | 1.07 (0.88—1.29) |
| 130<140 | Reference | Reference | Reference |
| 140<150 | 1.15 (1.02 – 1.30) | 1.10 (0.99—1.23) | 1.08 (0.90—1.29) |
| 150<160 | 1.31 (1.14 – 1.50) | 1.24 (1.10—1.40) | 1.17 (0.95—1.44) |
| ≥160 | 1.24 (1.30 – 1.71) | 1.40 (1.23—1.58) | 1.25 (1.00—1.55) |
| **Heart failure hospitalization**  Systolic Blood pressure (mmHg) | **Adjusted HR (95% CI)**  **eGFR<30**  **Mean SBP first year**  **N=16,822** | **Adjusted HR (95% CI)**  **Albuminuria adjusted in all**  **Mean SBP**  **N=23,317** | **Adjusted HR^#^ (95% CI)**  **Restricted to patients with albuminuria measurements**  **Mean SBP**  **N=12,649** |
| 100<120 | 1.04 (0.92 – 1.18) | 1.03 (0.92—1.14) | 1.11 (0.93—1.34) |
| 120<130 | 0.94 (0.85 – 1.05) | 0.96 (0.87—1.05) | 0.92 (0.78—1.09) |
| 130<140 | Reference | Reference | Reference |
| 140<150 | 1.07 (0.92 – 1.18) | 1.06 (0.97—1.16) | 1.16 (0.99—1.35) |
| 150<160 | 1.09 (0.97 – 1.22) | 1.06 (0.97—1.18) | 1.11 (0.93—1.32) |
| ≥160 | 1.39 (1.23 – 1.56) | 1.32 (1.19—1.47) | 1.36 (1.14—1.62) |
| **Stroke**  Systolic Blood pressure (mmHg) | **Adjusted HR (95% CI)**  **eGFR<30**  **Mean SBP first year**  **N=16,822** | **Adjusted HR (95% CI)**  **Albuminuria adjusted in all**  **Mean SBP**  **N=23,317** | **Adjusted HR^#^ (95% CI)**  **Restricted to patients with albuminuria measurements**  **Mean SBP**  **N=12,649** |
| 100<120 | 0.92 (0.72 – 1.19) | 1.02 (0.85—1.24) | 0.97 (0.67—1.40) |
| 120<130 | 0.86 (0.69 – 1.06) | 0.98 (0.83—1.15) | 1.02 (0.76—1.36) |
| 130<140 | Reference | Reference | Reference |
| 140<150 | 0.93 (0.77 – 1.13) | 0.94 (0.81—1.10) | 0.81 (0.62—1.08) |
| 150<160 | 1.25 (1.02 – 1.53) | 1.08 (0.92—1.26) | 0.93 (0.69—1.26) |
| ≥160 | 1.58 (1.31 – 1.90) | 1.39 (1.18—1.65) | 1.16 (0.86—1.57) |
| *Hazard ratios were estimated using multivariable-adjusted models with 130–<140 mmHg as the reference SBP category. The eGFR <30 ml/min/1.73 m² analysis was restricted to patients with advanced CKD. In the albuminuria-adjusted analysis, missing albuminuria was treated as a separate category. The complete-case albuminuria analysis was restricted to patients with available albuminuria measurements. These analyses were performed to assess robustness across kidney disease severity and the potential influence of albuminuria. Models were adjusted for age at baseline (restricted cubic spline), sex, diabetes, history of myocardial infarction, peripheral arterial disease, atrial fibrillation, heart failure, plasma albumin, phosphate, eGFR, diastolic blood pressure (restricted cubic spline), current use of diuretics, ACEi/ARB, betablockers*  *In the complete-case analysis, albuminuria was modelled continuously using restricted cubic splines.* | | | |

**Table S7** Comparison of characteristics between participants in the main analysis (n=23,317) vs those who excluded from analyses N=4812 and even those whom excluded due to SBP<100 mmHg (N=244)

| **Clinical characteristics** | **Main analysis participants**  **N=23,317** | **Excluded from analyses**  **N=4812** | **Excluded due to SBP<100 mmHg**  **N=244** |
| --- | --- | --- | --- |
| Age, median (IQR) | 71.8 (62.7 – 79.2) | 75.2 (64 – 82) | 73 (66–81) |
| Female % | 36.5 | 35.2 | 36.1 |
| **Comorbidities *(%)*** |  | |  |
| Myocardial infarction | 22.6 | 31.7 | 46.3 |
| Diabetes mellitus | 39.9 | 43.8 | 41.8 |
| Heart failure | 22.8 | 39.5 | 59.8 |
| Cerebrovascular disease | 17.1 | 20.6 | 20.1 |
| Atrial fibrillation | 17.4 | 27.9 | 44.3 |
| **Clinical data**  *Median (IQR)* |  | |  |
| BMI | 27.4 (24.2 - 31.2) | 26.0 (22.9 – 29.9) | 25.8 (22.2–29.3) |
| eGFR | 24.7 (18.5 - 31.2) | 18.9 (12.5 – 26.8) | 27.3 (20.6–34.8) |
| SBP | 138.0 (126.7 - 150.0) | 136.7 (120.0 – 151.3) | 95.0 (90.0–97.0) |
| DBP | 77.5 (70.0 – 83.0) | 75.0 (66 – 82.5) | 60.0 (55.0–64.0) |
| Charlson comorbidity index | 4.0 (2.0 - 6.0) | 5.0 (3.0 - 7.0) | 5.0 (4.0–7.0) |
| **Routine laboratory data**  *Median (IQR)* |  | |  |
| Albumin | 37.0 (34.0 - 40.0) | 35.0 (32.0 - 39.0) | 36.0 (37.0–39.0) |
| Hemoglobin | 123 (112 - 134) | 116 (105 - 127) | 121 (112–131) |
| Potassium | 4.4 (4.0 - 4.7) | 4.4 (4.0 - 4.8) | 4.4 (3.9–4.7) |
| Cholestrol | 4.8 (4.0 - 5.7) | 4.5 (3.7 - 5.5) | 4.1 (3.2 – 5.0) |
| ACR | 29.0 (5.3 – 125.0) | 46.6 (8.6–218.5) | 6.8 (2.1 – 22.1) |
| **Current medication**  *(%)* |  | |  |
| ACEi/ARB | 69.6 | 61.8 | 59.8 |
| CCB | 56.7 | 57.5 | 12.3 |
| Beta-Blockers | 62.7 | 68.5 | 66.8 |
| Insulin | 27.2 | 29.1 | 25.8 |
| Diuretics | 65.3 | 76.4 | 74.2 |
| ESA | 5.4 | 6.9 | 5.8 |
| Oral diabetes medications | 10.8 | 8.9 | 8.6 |
| *CI (confidence interval). N (Number). eGFR (estimated glomerular filtration rate). SBP Systolic Blood Pressure. DBP Diastolic Blood Pressure. ACEi (angiotensin converting enzyme inhibitors). ARB (angiotensin 2 receptor blockers). IQR (interquartile range). SD (standard deviation). BMI (body mass index). MRA (Mineralocorticoid Receptor Antagonist). n/a (not applicable). DM (Diabetes Mellitus). CHF (Congestive Heart Failure). CVD (Cerebrovascular Disease). ACR (albumin Creatinine Ratio). ESA (Erythropoetin Stimulating agents).*  *Values are presented as n (%) or median (interquartile range). Included patients were those eligible for the primary analysis after application of study inclusion and exclusion criteria. Excluded patients are shown to describe potential selection introduced by cohort restrictions.*  *Patients with SBP <100 mmHg are presented separately because this group was evaluated in sensitivity analyses.* | | | |

**Table S8** Sensitivity analyses for MACE, MACE or death, and all-cause mortality using alternative baseline definitions

| **Major adverse cardiovascular events (MACE)**  Systolic Blood pressure (mmHg) | **Adjusted HR (95% CI)**  **Total cohort**  **First SBP**  **N=28,113** | **Adjusted HR (95% CI)**  **6m baseline period**  **Mean 6m SBP**  **N=18,648** | **Adjusted HR (95% CI)**  **12-month baseline period, min one follow-up**  **Mean SBP**  **N=15,668** |
| --- | --- | --- | --- |
| 100<120 | 1.09 (1.02 – 1.17) | 0.96 (0.88-1.04) | 0.96 (0.87-1.06) |
| 120<130 | 0.98 (0.92 – 1.04) | 0.96 (0.89-1.04) | 0.94 (0.87-1.03) |
| 130<140 | Reference | Reference | Reference |
| 140<150 | 1.10 (1.04 – 1.17) | 1.06 (0.99 – 1.14) | 1.07 (0.99-1.15) |
| 150<160 | 1.11 (1.03 – 1.19) | 1.09 (1.01 – 1.19) | 1.09 (1.00-1.19) |
| ≥160 | 1.34 (1.25– 1.44) | 1.26 (1.16 – 1.36) | 1.41 (1.29-1.54) |
| **Major adverse cardiovascular events + death (MACE and Death)**  Systolic Blood pressure (mmHg) | **Adjusted HR (95% CI)**  **Total cohort**  **First SBP**  **N=28,113** | **Adjusted HR (95% CI)**  **6m baseline period**  **Mean 6m SBP**  **N=18,648** | **Adjusted HR (95% CI)**  **12-month baseline period, min one follow-up**  **Mean SBP**  **N=15,668** |
| 100<120 | 1.12 (1.05 – 1.19) | 0.96 (0.88-1.04) | 0.99 (0.86 – 1.15) |
| 120<130 | 1.00 (0.94 – 1.06) | 0.91 (0.85–0.97) | 0.99 (0.87-1.13) |
| 130<140 | Reference | Reference | Reference |
| 140<150 | 1.06 (1.01 – 1.12) | 1.03 (0.97 – 1.10) | 1.11 (0.98-1.26) |
| 150<160 | 1.09 (1.02 – 1.15) | 1.09 (1.02 – 1.17) | 1.19 (1.03-1.37) |
| ≥160 | 1.28 (1.21 – 1.36) | 1.29 (1.20 – 1.39) | 1.47 (1.28-1.68) |
| **All-cause mortality**  Systolic Blood pressure (mmHg) | **Adjusted HR (95% CI)**  **Total cohort**  **First SBP**  **N=28,113** | **Adjusted HR (95% CI)**  **6m baseline period**  **Mean 6m SBP**  **N=18,648** | **Adjusted HR (95% CI)**  **12-month baseline period, min one follow-up**  **Mean SBP**  **N=15,668** |
| 100<120 | 1.16 (1.08 – 1.25) | 1.00 (0.91 – 1.10) | 0.99 (0.91-1.07) |
| 120<130 | 1.00 (0.93 – 1.07) | 0.99 (0.91 – 1.07) | 0.96 (0.89 – 1.03) |
| 130<140 | Reference | Reference | Reference |
| 140<150 | 1.01 (0.95 – 1.08) | 1.00 (0.92—1.08) | 1.04 (0.97 – 1.11) |
| 150<160 | 1.04 (0.97 – 1.12) | 1.07 (0.98—1.17) | 1.07 (0.99-1.16) |
| ≥160 | 1.23 (1.14 – 1.32) | 1.14 (1.04—1.24) | 1.33 (1.23-1.44) |
| *Hazard ratios were estimated using multivariable-adjusted models with 130–<140 mmHg as the reference SBP category. The total cohort analysis used the first available SBP measurement. The 6-month and 12-month landmark analyses used mean SBP during the respective baseline period among patients alive and under follow-up at the landmark time. Estimates are shown to assess the robustness of the primary findings to alternative exposure definitions and landmark periods.* | | | |

**Table S9** Sensitivity analyses for individual components of MACE using alternative baseline definitions

| **Cardiovascular death**  Systolic Blood pressure (mmHg) | **Adjusted HR (95% CI)**  **Total cohort**  **First SBP**  **N=28,113** | **Adjusted HR (95% CI)**  **6m baseline period**  **Mean 6m SBP**  **N=18,648** | **Adjusted HR (95% CI)**  **12-month baseline period, min one follow-up**  **Mean SBP**  **N=15,668** |
| --- | --- | --- | --- |
| 100<120 | 1.09 (0.95– 1.27) | 0.96 (0.82—1.13) | 1.02 (0.85-1.22) |
| 120<130 | 0.96 (0.84 – 1.10) | 0.96 (0.83—1.10) | 0.95 (0.81-1.11) |
| 130<140 | Reference | Reference | Reference |
| 140<150 | 1.14 (1.00 – 1.28) | 1.05 (0.91—1.20) | 1.11 (0.96-1.28) |
| 150<160 | 1.05 (0.91 – 1.22) | 1.14 (0.98—1.31) | 1.04 (0.88-1.23) |
| ≥160 | 1.29 (1.12 – 1.48) | 1.13 (0.98—1.31) | 1.27 (1.07-1.50) |
| **Myocardial infarction**  Systolic Blood pressure (mmHg) | **Adjusted HR (95% CI)**  **Total cohort**  **First SBP**  **N=28,113** | **Adjusted HR (95% CI)**  **6m baseline period**  **Mean 6m SBP**  **N=18,648** | **Adjusted HR (95% CI)**  **12-month baseline period, min one follow-up**  **Mean SBP**  **N=15,668** |
| 100<120 | 0.74 (0.62 – 0.89) | 0.65 (0.53—0.80) | 0.67 (0.53-0.85) |
| 120<130 | 0.98 (0.84 – 1.13) | 0.83 (0.71—0.99) | 0.99 (0.83-1.18) |
| 130<140 | Reference | Reference | Reference |
| 140<150 | 1.23 (1.08 – 1.40) | 0.93 (0.80—1.08) | 1.16 (0.99-1.36) |
| 150<160 | 1.22 (1.05 – 1.42) | 1.06 (0.90—1.26) | 1.08 (0.89-1.31) |
| ≥160 | 1.74 (1.51 – 2.00) | 1.40 (1.19—1.64) | 1.74 (1.46-2.09) |
| **Coronary heart disease**  Systolic Blood pressure (mmHg) | **Adjusted HR (95% CI)**  **Total cohort**  **First SBP**  **N=28,113** | **Adjusted HR (95% CI)**  **6m baseline period**  **Mean 6m SBP**  **N=18,648** | **Adjusted HR (95% CI)**  **12-month baseline period, min one follow-up**  **Mean SBP**  **N=15,668** |
| 100<120 | 0.79 (0.67 – 0.92) | 0.90 (0.79—1.03) | 0.90 (0.77-1.05) |
| 120<130 | 1.01 (0.89 – 1.15) | 1.04 (0.92—1.17) | 1.04 (0.92-1.18) |
| 130<140 | Reference | Reference | Reference |
| 140<150 | 1.17 (1.04 – 1.32) | 1.10 (0.98—1.23) | 1.14 (1.01-1.29) |
| 150<160 | 1.26 (1.10 – 1.44) | 1.08 (0.95—1.23) | 1.30 (1.13-1.49) |
| ≥160 | 1.48 (1.28 – 1.70) | 1.28 (1.13—1.45) | 1.50 (1.30-1.74) |
| **Heart failure hospitalization**  Systolic Blood pressure (mmHg) | **Adjusted HR (95% CI)**  **Total cohort**  **First SBP**  **N=28,113** | **Adjusted HR (95% CI)**  **6m baseline period**  **Mean 6m SBP**  **N=18,648** | **Adjusted HR (95% CI)**  **12-month baseline period, min one follow-up**  **Mean SBP**  **N=15,668** |
| 100<120 | 0.99 (0.87 – 1.13) | 0.97 (0.88—1.09) | 0.98 (0.87—1.11) |
| 120<130 | 0.88 (0.79 – 0.99) | 0.94 (0.85—1.05) | 0.94 (0.84—1.05) |
| 130<140 | Reference | Reference | Reference |
| 140<150 | 1.12 (1.01 – 1.25) | 1.03 (0.93—1.14) | 1.08 (0.98—1.20) |
| 150<160 | 1.18 (1.05– 1.33) | 1.02 (0.92—1.14) | 1.09 (0.97—1.23) |
| ≥160 | 1.49 (1.32 – 1.67) | 1.29 (1.16—1.43) | 1.43 (1.27—1.61) |
|  |  |  |  |
| **Stroke**  Systolic Blood pressure (mmHg) | **Adjusted HR (95% CI)**  **Total cohort**  **First SBP**  **N=28,113** | **Adjusted HR (95% CI)**  **6m baseline period**  **Mean 6m SBP**  **N=18,648** | **Adjusted HR (95% CI)**  **12-month baseline period, min one follow-up**  **Mean SBP**  **N=15,668** |
| 100<120 | 1.00 (0.82 – 1.23) | 0.96 (0.79—1.18) | 1.02 (0.82—1.26) |
| 120<130 | 1.01 (0.85 – 1.20) | 0.99 (0.83—1.17) | 0.94 (0.79—1.13) |
| 130<140 | Reference | Reference | Reference |
| 140<150 | 0.97 (0.82 – 1.14) | 1.10 (0.94—1.29) | 0.97 (0.82—1.15) |
| 150<160 | 1.15 (0.97 – 1.38) | 1.11 (0.93—1.33) | 0.97 (0.80—1.18) |
| ≥160 | 1.43 (1.20 – 1.71) | 1.33 (1.22—1.57) | 1.39 (1.15—1.68) |
| *Hazard ratios were estimated using multivariable-adjusted models with 130–<140 mmHg as the reference SBP category. The total cohort, 6-month baseline, and 12-month baseline analyses used the exposure definitions described in Table S6. Individual MACE components were analyzed.* separately. Estimates are presented to assess whether the association between SBP and MACE was driven by specific cardiovascular outcomes. *Models were adjusted for age at baseline (restricted cubic spline), sex, diabetes, history of myocardial infarction, peripheral arterial disease, atrial fibrillation, heart failure, plasma albumin, phosphate, eGFR, diastolic blood pressure (restricted cubic spline), current use of diuretics, ACEi/ARB, betablockers* | | | |

**Table S10**. Sensitivity analysis with kidney replacement therapy as time-dependent covariate

| Systolic Blood pressure (mmHg) | **Major cardiovascular events (MACE)**  HR (95% confidence interval) | **All-cause mortality**  HR (95% confidence interval) | **Major cardiovascular events or all-cause mortality**  HR (95% confidence interval) |
| --- | --- | --- | --- |
| 100<120 | 0.96 (0.89-1.05) | 0.99 (0.91—1.08) | 0.97 (0.90-1.04) |
| 120<130 | 0.94 (0.87 – 1.00) | 0.92 (0.85—0.99) | 0.94 (0.80-1.00) |
| 130<140 | Reference | Reference | Reference |
| 140<150 | 1.05 (0.98 – 1.13) | 0.96 (0.90—1.03) | 1.01 (0.95-1.07) |
| 150<160 | 1.12 (1.04 – 1.21) | 1.05 (0.97—1.14) | 1.09 (1.02-1.16) |
| ≥160 | 1.36 (1.26 – 1.48) | 1.22 (1.13—1.33) | 1.27 (1.18-1.36) |
|  |  |  |  |

*Hazard ratios (HR) were estimated using multivariable-adjusted models with 130–<140 mmHg as the reference SBP category. Models were adjusted for age at baseline (restricted cubic spline), sex, diabetes, history of myocardial infarction, peripheral arterial disease, atrial fibrillation, heart failure, plasma albumin, phosphate, eGFR, diastolic blood pressure (restricted cubic spline), current use of diuretics, ACEi/ARB, betablockers, and kidney replacement therapy as a time dependent covariate.*

**Figure S1.** Description of study methods for index visit and follow‑up (timeline).

**Figure S2. Association between systolic blood pressure and MACE including non‑cardiovascular death.**

**Figure S3.** Association between systolic blood pressure and myocardial infarction.

**Figure S4.** Association between systolic blood pressure and cardiovascular death.

**Figure S5.** Association between systolic blood pressure and coronary arterial disease events.

**Figure S6.** Association between systolic blood pressure and heart failure.

**Figure S7.** Association between systolic blood pressure and stroke.
